# Synergistic effects of warming and elevated CO_2_ intensify drought impacts on grassland carbon and water fluxes

**DOI:** 10.1126/sciadv.aea8988

**Published:** 2026-06-26

**Authors:** Maud Tissink, Jesse Radolinski, Johannes Cunow, Andreas Schaumberger, Lumnesh S. K. Joseph, Mirco Migliavacca, Markus Reichstein, Michael Bahn

**Affiliations:** ^1^ Department of Ecology, Universität Innsbruck, Sternwartestraße 15, A-6020 Innsbruck, Austria.; ^2^ College of Agriculture, Arkansas State University, Jonesboro, AR 72467, USA.; ^3^ Division of Agriculture, University of Arkansas, Little Rock, AR 72207, USA.; ^4^ Agricultural Research and Education Centre (AREC), Raumberg-Gumpenstein, Raumberg 38, A-8952 Irdning, Austria.; ^5^ European Commission, Joint Research Centre (JRC), Ispra, Italy.; ^6^ Department of Biogeochemical Integration, Max Planck Institute for Biogeochemistry, Jena, Germany.; ^7^ Integrative Center for Biodiversity Research (iDIV), Leipzig, Germany.

## Abstract

Understanding how rising carbon dioxide (CO_2_), climate warming, and drought interact to alter ecosystem functioning is critical for predicting future ecosystem resilience and land-atmosphere feedbacks. Using a long-term grassland experiment, we tested the individual and combined effects of elevated CO_2_ (eCO_2_), warming, and drought on ecosystem functional properties, including water vapor and CO_2_ fluxes, and water- and carbon-use efficiency (WUE and CUE, respectively). While eCO_2_ and warming had limited effects individually, their interaction synergistically amplified ecosystem respiration and drought impacts on most properties. Under ambient rainfall, this interaction did not reduce net ecosystem productivity (NEP), as higher respiration was counterbalanced by periodic increases in CO_2_ uptake. However, combined eCO_2_ and warming worsened the negative drought effects on CO_2_ fluxes and WUE and on postdrought evapotranspiration and CUE. This produced the lowest seasonal NEP, involving a fourfold stronger decline than under ambient drought. Our findings highlight that future climate conditions may decrease the capacity of drought-exposed ecosystems for water use and net carbon uptake.

## Teaser

Warmer, CO_2_-rich conditions synergistically amplify drought effects, prolong recovery, and reduce net carbon uptake capacity.

## INTRODUCTION

Rising atmospheric carbon dioxide (CO_2_), climate warming, and increasing drought are altering the functioning of ecosystems globally (*1*). However, it remains unclear how these factors—individually and combined—will affect ecosystem functional properties related to water and CO_2_ fluxes [sensu (*2*)] over time. These properties include ecosystem fluxes of water and CO_2_, water-use efficiency (WUE; the maximum CO_2_ gained per unit of water lost) and carbon-use efficiency [CUE; the maximum proportion of carbon (C) retained after respiration]. Together, they underpin an ecosystem’s capacity to maintain structure and function, governing its C stock trajectory and responses to disturbances such as heatwaves and drought (*2*, *3*). Despite this significance, major knowledge gaps persist regarding (i) how future warmer, CO_2_-enriched conditions alter ecosystem functional properties, including under conditions of drought (*3*–*5*); (ii) whether these future conditions will shift the balance between immediate (“concurrent”) and delayed (“legacy”) drought effects (*5*–*7*); and (iii) whether the combined effects of such global change factors are primarily additive or nonadditive (*8*–*12*). These uncertainties limit our ability to anticipate future shifts in water and C cycling, creating challenges for managing ecosystem stability and function (*3*).

Meta-analyses have suggested that effects of elevated CO_2_ (eCO_2_) and warming on ecosystem functional properties are predominantly additive when these factors combine (*8*, *9*). In this additive scenario, the impact of combined eCO_2_ and warming is approximately equal to the sum of their effects alone, which arise through distinct ecophysiological pathways. Under eCO_2_, plants typically reduce their stomatal conductance, which can improve WUE and conserve soil moisture (*13*–*16*). Leaf-scale photosynthesis may also increase, creating potential for increased gross primary productivity (GPP), net ecosystem productivity (NEP), and plant growth (*13*, *17*). Most commonly, however, this potential is constrained by nutrient or water limitation (*18*–*21*) or, in the case of NEP, offset by increased ecosystem respiration (R_eco_) (*22*). Warming, by contrast, may lead to reduced ecosystem WUE by increasing atmospheric water demand [vapor pressure deficit (VPD)] and promoting water loss (e.g., through soil evaporation) (*23*, *24*), although plants may close their stomata to limit transpiration (*16*, *25*). By increasing metabolic rates, warming can also increase R_eco_ and GPP, albeit with variable outcomes for NEP (*8*, *9*, *11*, *26*). In recent years, it has been questioned whether effects of eCO_2_ and warming on C cycling are truly additive (*12*). Another possibility is that nonadditive interactions, which can be smaller than additive (antagonistic) or larger than additive (synergistic), are remaining undetected unless they exceed the combined uncertainty of single-factor effects in size (*12*). This leaves substantial uncertainty for predicting ecosystem fluxes under future warmer, CO_2_-enriched conditions, especially for ecosystem water use [evapotranspiration (ET)] and regulation (WUE), which remain poorly constrained despite the projection that many regions will experience increased water limitation (*1*, *27*).

The third global change factor, drought, distinctly disrupts water and CO_2_ fluxes across terrestrial ecosystems—altering WUE and CUE—through both concurrent and legacy effects (*4*, *6*, *7*, *26*, *28*–*31*). As soil dries during drought, plants typically reduce stomatal aperture, causing ET to decline and GPP to decrease (*31*). Increasing sensitivity of stomatal conductance to evaporative demand can subsequently improve ecosystem WUE by limiting ET more than GPP, although this may not fully offset declines in WUE under very high evaporative demand (*4*, *25*, *32*–*34*). Eventually, coordinated whole-plant, microbial, and rhizosphere responses typically cause GPP to decline more sharply than R_eco_ (*18*, *35*, *36*), resulting in reduced NEP and CUE. Postdrought, legacy effects arising from persistent structural, physical, and physiological changes may continue to alter these ecosystem fluxes and their balance (*5*, *7*, *30*). While their magnitude and duration vary depending on factors like ecosystem type and drought severity (*5*, *29*, *37*–*40*), fluxes typically recover to undisturbed levels within a year (*30*, *41*, *42*). Previous work has highlighted potential for future warmer, CO_2_-enriched conditions to interact with the effects of drought (*30*, *39*, *40*, *43*). For example, concurrent drought impacts on ecosystem fluxes may be alleviated under eCO_2_ if down-regulated plant activity limits water loss and slows the drying of soil (*19*, *20*, *44*). Conversely, drought impacts may be intensified in a warmer climate (*45*, *46*) where ecosystem water loss and respiration are increased (*23*). The effect of drought under a combination of eCO_2_ and warming has, however, rarely been tested. The few existing studies suggest that drought effects on GPP and NEP may be alleviated under such future conditions (*40*, *43*). Yet, corresponding changes in ecosystem water use and WUE and the interactions among effects of these three global change factors (additive and nonadditive) have received almost no attention at all (*4*, *39*). It also remains unclear how eCO_2_ and warming shape drought legacy effects on ecosystem functional properties—whether they alter cumulative drought impacts, whether they modify recovery rates [see evidence for faster GPP recovery under warmer, CO_2_-enriched conditions (*40*, *43*)], or a combination of both (*37*). Consequently, the overall impact of future conditions on ecosystem drought responses remains highly uncertain and challenging to predict.

Here, we assessed how individual and combined treatments of eCO_2_, warming, and drought affect ecosystem functional properties related to water and CO_2_ fluxes in a long-term grassland experiment. Treatments simulated projected future climate scenarios, namely, late-21st-century increased atmospheric CO_2_ [+300 parts per million (ppm)] and warming (+3°C canopy surface temperature) and severe natural drought (*1*, *47*). “Drought” treatments involved the exclusion of summer rainfall under ambient (termed “ambient drought”) and warmer, CO_2_-enriched conditions (“future drought”). We derived ecosystem functional properties (ET_sat_, GPP_sat_, WUE_sat_, NEP_sat_, R_eco_, and CUE_sat_, where subscript “sat” indicates properties based on light-saturated GPP) from chamber-based flux measurements at peak daily photosynthetic activity over two growing seasons. Grasslands are typically sensitive to climate variability, yet responses to eCO_2_, warming, and drought remain poorly resolved in mesic systems managed by regular mowing and fertilization (*9*, *10*), which are common across Europe and the focus of the present study. We addressed two key questions: (i) How do eCO_2_ and warming, individually and combined, affect these ecosystem functional properties? (ii) How do future warmer, CO_2_-enriched conditions alter these ecosystem functional properties during and after drought? For the first question, we anticipated that eCO_2_ would result in negligible change in ecosystem fluxes but improve WUE_sat_ and reduce CUE_sat_, while warming would promote ecosystem fluxes but reduce WUE_sat_ and CUE_sat_. Expecting additive effects of eCO_2_ and warming (*8*, *9*), we hypothesized that (i) the combination of warmer and CO_2_-enriched conditions would promote ecosystem fluxes and lead to negligible change in WUE_sat_ and reduced CUE_sat_. For the second question, we hypothesized that (ii) ambient drought would lead to reduced water and CO_2_ fluxes, improved WUE_sat_, and reduced CUE_sat_. In addition, we hypothesized that (iii) under warmer, CO_2_-enriched conditions, drought effects would intensify, but recovery would be faster. Addressing these hypotheses will depict ecosystem functioning under current and future scenarios of climate and drought—insight urgently needed as Earth’s climate becomes increasingly drought-prone.

## RESULTS

### eCO_2_ and warming synergistically amplify R_eco_ and drought impacts

**Fig. 1.**
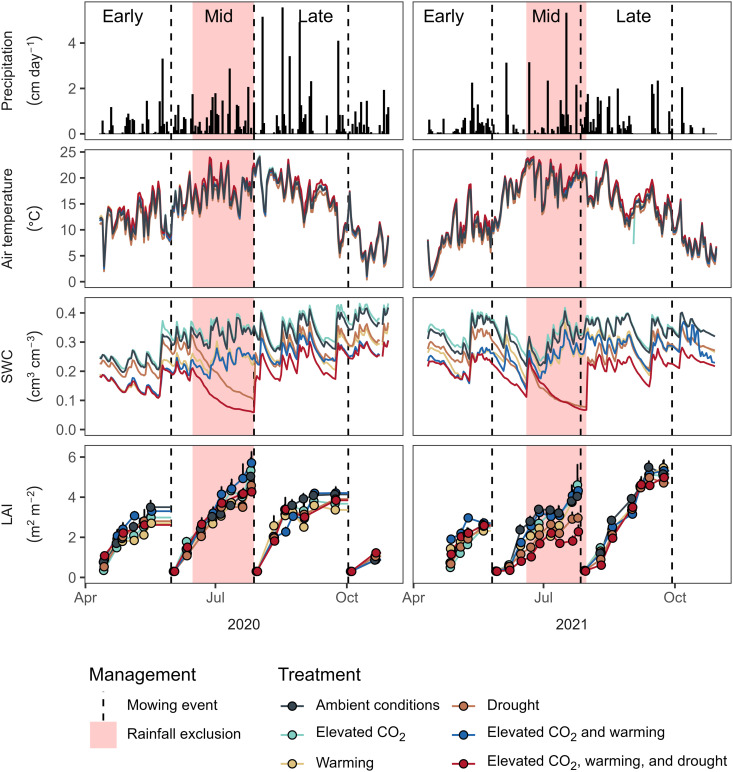
Growing-season conditions across global change treatments. Daily precipitation, air temperature, SWC (depth-weighted from 0 to 25 cm), and LAI over two growing seasons. Mowing events occurred three times per year, defining the early, mid-, and late periods of each growing season. Treatments include individual and combined applications of eCO_2_ (+300 ppm), warming (+3°C canopy surface temperature), and drought (summer rainfall exclusion, ended with 40-mm rewetting). Colored lines indicate mean treatment values over time; points show the treatment means for individual sampling campaigns (treatment replicate range between 3 and 8; see the “Experimental design” section) with error bars indicating standard errors.

**Fig. 2.**
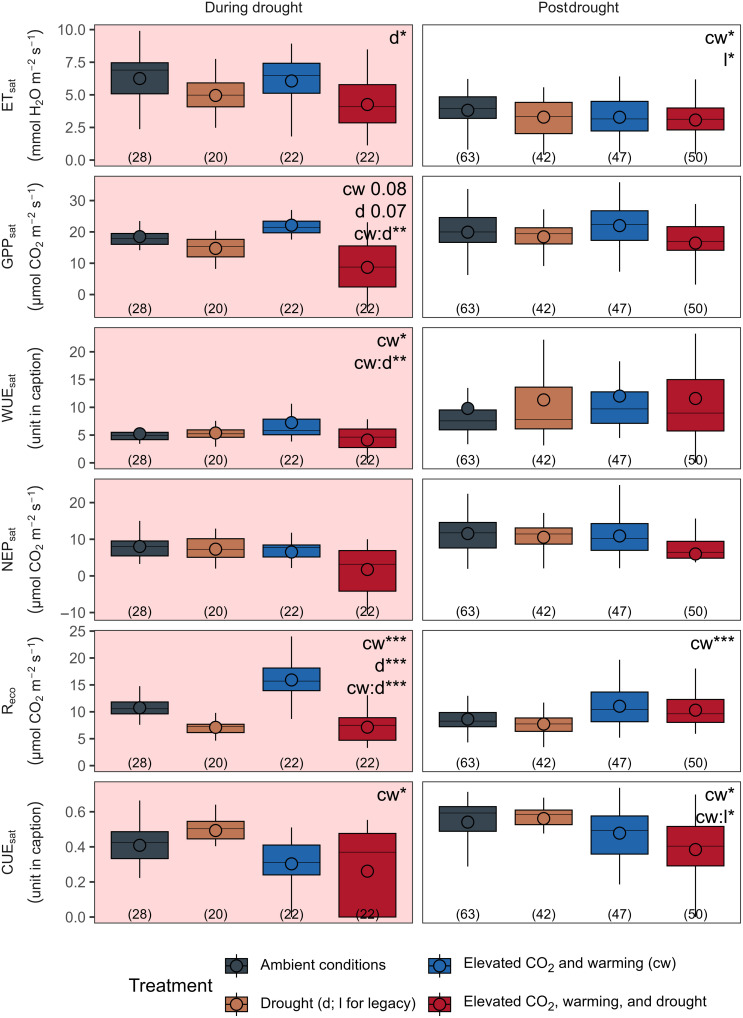
Ecosystem functional properties related to water and CO_2_ fluxes across individual and combined treatments of future conditions and drought. Future conditions refer to a combination of eCO_2_ and warming (individual effects: fig. S3), with separate consideration of functional properties during and after drought. Lower and upper boundaries of the box plots indicate the 25th and 75th quartiles of replicate samples across treatments over two growing seasons, center lines indicate median values, and whiskers indicate 1.5 times the interquartile range (*n* is reported in brackets below each box; source data: fig. S2). Points inside boxes are the estimated means from linear mixed models with treatments as fixed effects, with random intercept effects for plot and date. Text indicates treatments and nonadditive interactions with a statistically significant effect (abbreviations in the figure legend; detailed statistics: table S2). Asterisks denote associated *P* values (***P < 0.001, **P < 0.01, *P < 0.05, and *P* < 0.1 reported as values). CUE is expressed as μmol CO_2_ NEP μmol^−1^ CO_2_ GPP; WUE is expressed as μmol CO_2_ kPa^−0.5^ mmol^−1^ H_2_O. The subscript “sat” indicates ecosystem functional properties associated with light-saturated GPP.

**Fig. 3.**
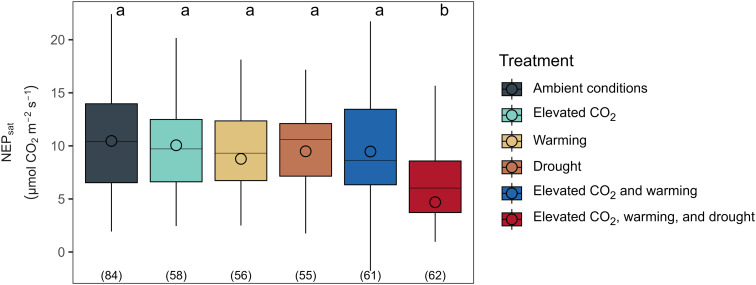
NEP across individual and combined treatments of eCO_2_, warming, and drought. Measurements were conducted over two growing seasons. Lower and upper boundaries of the box plots indicate the 25th and 75th quartiles of replicate samples across treatments over two growing seasons, center lines indicate median values, and whiskers indicate 1.5 times the interquartile range (*n* is reported in brackets below each box; source data: fig. S2). Points inside boxes are the estimated means from linear mixed models with treatments as fixed effects, with random intercept effects for plot and date. Distributions not sharing any letter differ at the 5% significance level (detailed statistics, also for other properties: table S1). The subscript “sat” indicates an association with light-saturated GPP.

Ecosystem functional properties, as well as canopy structure [i.e., height and leaf area index (LAI)] and hydroclimatic conditions, differed notably across individual and combined treatments of eCO_2_, warming, and drought over two growing seasons (Figs. 1 to 3, figs. S2 to S4, and tables S1 and S2). While we adopt a conventional significance threshold (α = 0.05) for identifying and discussing these treatment differences, we also report more limited evidence (α = 0.1) to support the interpretation of coordinated ecosystem responses. Treatment differences were most pronounced during mid-seasons, defined as the period between a first and second mowing event (vegetation cut followed by fertilization to compensate for nutrient loss). During this time, temperature and VPD were the highest, and rainfall exclusion reduced annual precipitation across drought treatments by 215 to 220 mm, or ~25% (Fig. 1). Conversely, differences were less prominent in the late season, when rainfall was no longer excluded across drought treatments and canopy greenness recovered (*48*). Notably, not only rainfall exclusions but also warming treatments tended to produce drier soil conditions, whereas any effects of eCO_2_ on soil water content (SWC) were inconsistent and comparatively small (fig. S1).

Across plots exposed to ambient rainfall, future conditions (eCO_2_ × warming) often produced the strongest responses in ecosystem functional properties among treatments, largely because effects of eCO_2_ and warming individually were typically small and/or statistically unclear (fig. S3; linear mixed-effect models; tables S1 and S2). Under this combined treatment, mid-season WUE_sat_ was almost 40% higher compared to ambient conditions, with limited evidence that this was driven primarily by an increase in GPP_sat_ (+19%; *P* = 0.08; Fig. 2). Moreover, warming amplified an increase in R_eco_ under eCO_2_ during the early-to-mid growing season (*P* = 0.01; Fig. 2 and fig. S3), more than doubling its size (from 15 to 36%; table S2), reducing CUE_sat_ (−16%), and preventing an increase in NEP_sat_ (Fig. 3). Notably, in this combined treatment, late-season ET_sat_ was reduced (−12%; table S2). By comparison, the individual treatments of eCO_2_ and warming elicited less pronounced responses of ecosystem functional properties, with only slight evidence for an increase in underlying WUE_sat_ under eCO_2_ (+19%; *P* = 0.07; fig. S3 and table S2).

Rainfall exclusions, on the other hand, had comparatively strong effects. The ambient drought treatment led to a ~20% decline in GPP_sat_ and ET_sat_ relative to plots continuously exposed to ambient rainfall, as well as a ~34% decline in R_eco_ (Fig. 2, fig. S2, and table S2). However, evidence for the GPP_sat_ reduction was limited (*P* = 0.07), and WUE_sat_, CUE_sat_, and NEP_sat_ exhibited no significant response (Fig. 2). Under warmer, CO_2_-enriched conditions, drought treatment effects on CO_2_ fluxes were amplified, with GPP_sat_ and R_eco_ declining 3 and 1.6 times more strongly, respectively, while WUE_sat_ was reduced (−21%; Fig. 2 and table S2). Toward the end of rainfall exclusion, these declines intensified as the SWC approached the permanent wilting point (10%), causing CUE_sat_ to approach zero (figs. S2 and S6). Following a 40-mm rewetting, which ended rainfall exclusion along with a mowing event (Fig. 1), ET_sat_ in the ambient drought treatment remained slightly suppressed (by 12%; Fig. 2 and table S2), while CO_2_ fluxes almost immediately recovered to levels observed in plots exposed to continuous ambient rainfall (fig. S4). A similar soil-moisture reduction occurred under warmer, CO_2_-enriched conditions; here, however, CUE_sat_ remained reduced by 30% for ~3 weeks postdrought. This dynamic resulted from a combination of an R_eco_ overshoot and a GPP_sat_ depression, neither of which were statistically significant individually (Fig. 2 and fig. S4).

Together, these functional responses to future drought conditions led to the lowest growing-season average of NEP_sat_ among treatments, inducing a four-times greater reduction compared to the ambient drought treatment (−63% versus −14%; Fig. 3 and table S1). Across combined treatments, nonadditive synergistic effects occurred about as often as additive effects. Overall, these drove the strongest treatment responses and became more pronounced as the number of co-occurring global change factors increased.

### Global change effects relate more to hydroclimatic than to canopy variables

**Fig. 4.**
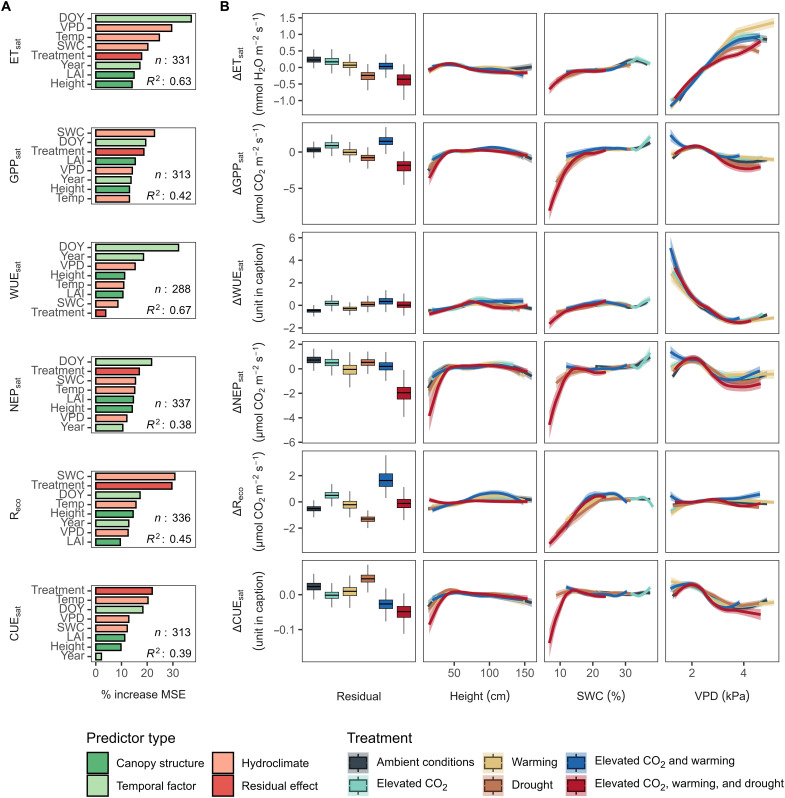
Importance and contributions of predictors to ecosystem functional properties related to water and CO_2_ fluxes under global change treatments. (**A**) Relative predictor importance of each property (“% increase in MSE”, where MSE is the mean square error) as determined using random forest models (*n* and *R*^2^ values reported). (**B**) Marginal contributions of key predictors to each property (all marginal effects: figs. S6 and S7), as indicated by SHAP. “Residual” effects represent any portion of the treatment effect not explained by predictors alone. Shaded areas indicate error ranges, calculated as standard deviations from 100 bootstrapped datasets (fig. S5). CUE is expressed as μmol CO_2_ NEP μmol^−1^ CO_2_ GPP; WUE is expressed as μmol CO_2_ kPa^−0.5^ mmol^−1^ H_2_O. The subscript “sat” indicates ecosystem functional properties associated with light-saturated GPP. Predictor abbreviations: Temp, temperature; DOY, day of year.

The studied ecosystem functional properties were moderately to well explained by predictors, including variables relating to hydroclimate, canopy structure, and time (*R*^2^ = 0.38 to 0.67; random forest regression; Fig. 4A), with results consistent across 100 bootstrapped datasets (fig. S5). The models included a categorical treatment term, referred to hereafter as the “residual” treatment effect, which reflected the portion of the treatment effect mediated by unmeasured factors (e.g., canopy greenness and nutrient availability). While random forest models inherently capture interactions among predictors, the use of Shapley Additive exPlanations (SHAP) to isolate marginal effects attributes treatment-predictor interactions to this residual term, together with any effects of unmeasured variables.

The strongest shifts in relationships between predictors and functional properties occurred in the future drought treatment. Here, the sensitivity of flux properties—both of water and CO_2_—to high VPD was comparatively low, especially toward the end of rainfall exclusions (Fig. 4B), reflecting reduced canopy conductance during periods of low SWC (fig. S8). Furthermore, GPP_sat_, NEP_sat_, and CUE_sat_ were comparatively low after mowing when the canopy height and LAI were reduced (Fig. 4B and figs. S6 and S7). Notably, this variation in canopy structure imposed by mowing in this managed system exceeds that observed in natural (nonmowed) grasslands (Fig. 1). The only other treatment in which relationships between predictors and functional properties shifted was the individual warming treatment, where water loss at high temperature and VPD increased (Fig. 4B and figs. S6 and S7). These treatment-dependent changes in predictor responses contributed to residual treatment effects in the models but did not fully account for the entire residuals, indicating that unmeasured factors also played a role. Other treatment effects with large residuals, such as increased R_eco_ and reduced CUE_sat_ under combined eCO_2_ and warming, further pointed to an influence of unmeasured factors.

**Fig. 5.**
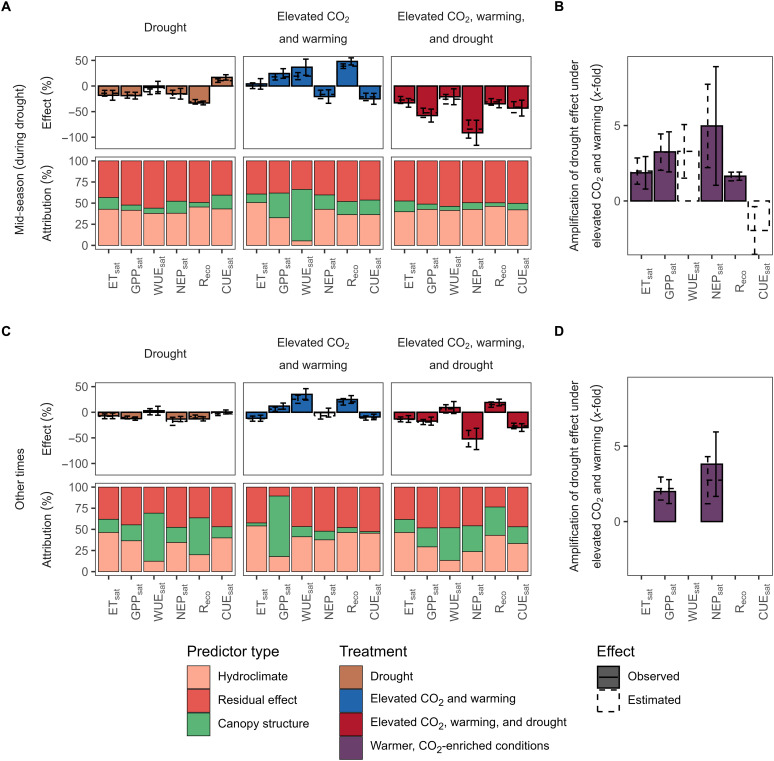
Size and drivers of the effects of global change treatments on ecosystem functional properties related to water and CO_2_ fluxes. Global change treatments refer to individual and combined treatments of future conditions (eCO_2_ and warming) and drought. Results are shown separately for (**A**) mid-seasons, when rainfall exclusions were applied across drought treatments, and (**C**) other periods (full two-season time course: Fig. 1). Upper panels show treatment effects (percentage difference from ambient conditions; error bars show standard errors), with right-hand facets (**B** and **D**) indicating any amplification (where error ranges do not overlap zero) of drought effects under future conditions. Effects are shown as observed (fig. S2) and as estimated by random forest regression models (Fig. 4). Lower panels show the attribution of treatment effects to shifts in hydroclimate, canopy structure, or other (“residual”) factors based on the relative predictor importance of these models (full model outputs, also for individual treatments of eCO_2_ and warming, and *n* values associated with each attribution: fig. S9). The subscript “sat” indicates ecosystem functional properties associated with light-saturated GPP.

Even with some uncertainty among drivers as indicated by residual terms, most treatment responses arose primarily from altered hydroclimatic conditions (canopy-level VPD and air temperature and SWC) rather than from canopy height and LAI (Fig. 5 and fig. S9). This was especially true under future drought conditions, where temperatures and VPD were higher and soils were drier than in other treatments, and a more limited role of reduced canopy height and LAI (Fig. 1 and fig. S1) was primarily played postdrought (Fig. 5). It also applied to the reduced fluxes under ambient drought conditions and to the increased R_eco_ under eCO_2_—including when combined with warming (Fig. 5). Only the enhanced mid-season WUE_sat_ under combined eCO_2_ and warming was predominantly associated with canopy structure (Fig. 5), coinciding with increased canopy height and LAI (Fig. 1 and fig. S1). Overall, hydroclimate mediated a larger share of global change effects on ecosystem functional properties than the canopy structure (and, thus, the mowing regime), especially for large effects under future drought conditions.

## DISCUSSION

This study explored how climate warming, eCO_2_, and drought may individually and collectively alter ecosystem functional properties related to water and CO_2_ fluxes (ET_sat_, GPP_sat_, WUE_sat_, NEP_sat_, R_eco_, and CUE_sat_). Our long-term experiment in a managed mesic grassland revealed two key findings: (i) Under warmer, CO_2_-enriched conditions, drought effects—especially concurrent ones but also legacies—were intensified and reshaped. (ii) Effects of combined global change factors often exceeded those of individual factors and frequently exhibited synergistic interactions, especially as the number of co-occurring factors increased. Overall, our findings reveal the functional pathways by which future drought conditions could markedly reduce ecosystem capacity for net C uptake.

### eCO_2_ and warming synergistically promote R_eco_

eCO_2_ is widely assumed to increase intrinsic WUE; however, its potential to promote GPP alongside R_eco_ in natural ecosystems—and thus to increase NEP—remains debated (*17*–*20*, *22*, *49*). In our study, we hypothesized that eCO_2_ (+300 ppm) would negligibly affect ecosystem fluxes but improve WUE_sat_ and CUE_sat_ such that GPP_sat_ would increase relative to both ET_sat_ and R_eco_. Contrary to this expectation, only R_eco_ responded clearly to eCO_2_, exhibiting a 15% increase relative to ambient conditions (fig. S3), consistent with responses of some other ecosystems (*9*, *11*). Given that this increase in R_eco_ was not clearly explained by moisture, temperature, or canopy variables alone (Figs. 4B and 5), and no concurrent increase was observed in GPP_sat_ (fig. S3) or plant biomass (*32*), the most likely driver is a stimulation of soil processes (*22*), given that both rhizosphere and microbial activities were reportedly enhanced (*50*, *51*). While many eCO_2_ studies report improved intrinsic WUE (*13*, *15*, *16*) and associated plant water savings (*14*), evidence for increased WUE_sat_ in our study was limited (*P* = 0.07; fig. S3), leaving uncertainty about the implications of eCO_2_ for coupled carbon-water fluxes in this managed mesic system. Notably, the 6 to 7 years of eCO_2_ preexposure in our experiment may explain some difference from studies with shorter or longer treatment durations (*11*). Overall, our results primarily support the notion that because eCO_2_ promotes R_eco_, increases in ecosystem C stocks in a future CO_2_-enriched climate may be minor (*18*, *52*).

Climate warming increases atmospheric water demand and metabolic rates and can diminish plant capacity to restrict water loss (*18*, *23*, *24*). Therefore, warming should promote ecosystem fluxes of water and CO_2_ while reducing WUE_sat_ and CUE_sat_. Yet, in our study, warming (+3°C canopy surface temperature) had no clear effect (fig. S3). This was unanticipated, as we would have expected higher growing-season ET_sat_ compared to ambient conditions, given the higher observed water use of the grassland at high VPD (Fig. 4B). We attribute this lack of ET_sat_ increase to a persistent reduction in rootzone soil moisture under warming (Fig. 1 and fig. S1), likely driven primarily by chronically increased evaporative demand (*23*, *24*). Modifications of soil hydraulic properties and pore water distribution under a warmer, drier rootzone (*39*) may have reinforced this pattern. As a result, our periodic ET_sat_ measurements likely reflect soil moisture–mediated (i.e., source-limited) constraints on water use (fig. S8C) (*32*, *53*). These dry conditions may in turn have limited any warming-induced increases in CO_2_ fluxes.

Given the individual effects of eCO_2_ and warming and their reportedly additive interactions in ecosystems (*8*, *9*), we expected that the combined treatment would amplify fluxes (ET_sat_, GPP_sat_, R_eco_, and NEP_sat_), reduce CUE_sat_, and leave WUE_sat_ largely unchanged (hypothesis 1). However, eCO_2_ and warming synergistically amplified R_eco_ and periodically increased WUE_sat_ by 40% when ET_sat_ was not reduced (fig. S3 and table S2). This synergistic effect on R_eco_, which is not clearly explained by predictors (Fig. 4B and figs. S6 and S7), likely occurred because warming amplified a process already stimulated by eCO_2_ (*43*). Although this process is probably soil related [as eCO_2_ did not increase plant biomass (*32*) but enhanced rhizosphere and microbial activities (*50*, *51*)] and is clearly sensitive to warming, its exact mechanism remains unclear and needs further investigation. Meanwhile, periods of enhanced WUE_sat_ coincided with increases in canopy height and LAI (Figs. 1 and 4B and figs. S6 and S7), which may have improved light interception (*4*) and light-use efficiency (*49*), with limited evidence suggesting that this was driven primarily by increased GPP_sat_ (*P* = 0.08). Toward the late season, the stimulating effects of warmer, CO_2_-enriched conditions on CO_2_ fluxes, WUE_sat_, and canopy structure diminished, while grassland water use declined (Figs. 2, 4B, and 5 and table S2). Thus, our findings support the notion that enhanced C dynamics in a future warmer, CO_2_-enriched climate may be undermined by water shortage (*27*) and may not realistically enhance ecosystem C stocks or yield (*54*).

### Warmer, CO_2_-enriched conditions intensify concurrent and legacy effects of drought

Drought commonly disrupts the functioning of terrestrial ecosystems, acting through both concurrent and legacy effects (*6*, *28*, *30*, *31*). During the event, ecosystems typically exhibit reduced fluxes of water and CO_2_, improved WUE, and reduced CUE (i.e., hypothesis 2) (*34*, *36*, *55*, *56*). In our study, the ambient drought treatment reduced ET_sat_, GPP_sat_, and R_eco_ by ~20 to 30%, as commonly observed across grasslands (*9*, *11*), although our evidence for the reduction of GPP_sat_ was limited (*P* = 0.07; Fig. 2). Hydroclimatic factors, especially the soil moisture decline, strongly explained these flux reductions (Fig. 5), which contradict global patterns in two ways (*34*, *55*, *56*). First, WUE_sat_ did not increase during ambient drought exposure (Fig. 2 and table S2), although the grassland reduced its water use at times when VPD was high and SWC reduced (fig. S8), consistent with stomatal regulation (*25*). This discrepancy arose despite our use of underlying WUE_sat_, which accounts more closely for a direct effect of VPD than the conventional metrics used in the reference studies (*34*, *55*), suggesting that any physiological gains in WUE under rainfall exclusion in the studied grassland might have been offset by the severity of ambient drought conditions (*25*, *33*). Second, CUE_sat_ remained stable as GPP_sat_ and R_eco_ declined in tandem (Fig. 2). This unusual coupling, observed during some nonsevere droughts (*36*), has been proposed to help ecosystems sustain net C uptake (*23*)—a notion supported by our findings (Fig. 3).

After precipitation returns following drought, ecosystem fluxes are generally thought to recover rapidly within several weeks to months (*30*, *41*, *42*). After rewetting in our study, however, previous drought exposure caused only modest reductions in canopy conductance and grassland water use (~10%; Fig. 2 and fig. S8B) despite reducing plant-available soil water (*39*), with CO_2_ fluxes and their ratio (CUE_sat_) remaining comparable to ambient conditions (fig. S4). This limited response could reflect a high functional resistance of the grassland, with no clear recovery signal following drought. Notably, this dynamic contrasts with earlier results from the site, where GPP_sat_, NEP_sat_, and CUE_sat_ declined during and after the first drought exposure despite similar reductions in SWC (*43*). Repeated exposure may therefore have modified the grassland’s drought response, most likely through legacies involving changes of soil properties, species adaptation, and shifts in community composition (*39*, *48*).

A primary aim of our study was to establish how the responses of ecosystem functional properties to drought shift under warmer, CO_2_-enriched conditions. Previous work indicates that warming intensifies drought effects (*45*, *46*), while eCO_2_ only partially alleviates them (*19*, *20*), and that these combined factors may speed postdrought recovery (*30*, *40*, *43*). Therefore, we expected that warmer, CO_2_-enriched conditions would amplify drought treatment effects but that greater impacts would be largely offset by enhanced recovery rates (hypothesis 3). Under these future conditions, drought-induced flux declines intensified (3-fold for GPP_sat_ and 1.6-fold for R_eco_; Figs. 2 and 5), while WUE_sat_ was reduced (−21%; table S2). Even though the decline in ET_sat_ did not intensify significantly (1.4-fold, *P* = 0.45; Figs. 2 and 5 and table S2), concurrent work demonstrates lower cumulative grassland water use at the same site (*32*, *39*), indicating that future drought conditions were more extreme overall. The sharp declines in ecosystem fluxes were primarily associated with extreme soil moisture depletion and, to a lesser extent, reductions in canopy height and LAI (Fig. 1 and fig. S1) and unmeasured factors (Figs. 4 and 5). This moisture depletion was more extreme than under ambient drought (Fig. 1), likely due to the added warming (which consistently reduced SWC across treatments; fig. S1), although additional pairwise treatments (drought × warming and drought × eCO_2_) would be needed to strictly disentangle the effect of eCO_2_. As soil became critically dry, with SWC approaching the permanent wilting point (10%; Fig. 4B), fluxes diminished, CUE_sat_ fell to zero, and NEP_sat_ turned negative (figs. S2 and S6), coinciding with a loss of greenness observed in a related phenocam study (*48*). Meanwhile, underlying WUE_sat_ was reduced under heightened VPD and temperature (Figs. 4B and 5 and fig. S4) despite stronger regulation of water loss by the grassland as its root access to water was reduced (fig. S8) (*39*). Together, the grassland’s capacity to avoid or tolerate drought appeared to be exceeded (*37*, *57*), contributing toward the lowest growing-season average of net C uptake capacity among treatments, representing a four-times larger decline in NEP_sat_ under future compared to ambient conditions of drought (Figs. 3 and 5 and table S1).

Following rainfall exclusion under warmer, CO_2_-enriched conditions, most functional properties (except R_eco_ and WUE_sat_) returned to levels observed in ambient-rainfall plots within the growing season (fig. S4). Recovery rates under these future drought conditions appeared higher than under ambient drought conditions (consistent with hypothesis 3), although limited postdrought impact on ambient properties calls into question whether there was much recovery to observe. Even so, our results indicate that higher recovery rates are unlikely to fully offset the intensified drought effects on ecosystem functional properties related to water and CO_2_ fluxes in a future warmer, CO_2_-rich climate.

The future drought treatment did have one distinct legacy effect: the reduction of CUE_sat_ (−30%; Fig. 2 and table S2), driven by an unexpected postdrought R_eco_ overshoot and GPP_sat_ suppression (fig. S4). This legacy lasted for 3 weeks—longer than an initial pulse of CO_2_ release triggered by rewetting dry soil [the “Birch effect” (*51*)]—and probably reflects a negative interaction between future drought conditions and mowing following rainfall exclusion (Figs. 1 and 4B). A previous study reporting modified flux responses to mowing under climatic extremes attributed these shifts to the greater sensitivity of rootzone moisture to evaporative demand when canopy cover is reduced (e.g., because of more efficient water vapor removal by wind) (*58*). Although this mechanism is consistent with the drier soil conditions observed under elevated VPD in the future drought treatment in our study (Fig. 1 and fig. S1), higher-resolution measurements would be needed to distinguish between this and further possible mechanisms. Even so, the observed interaction supports the notion that productivity benefits of grassland mowing may decline under future extremes (*58*), contributing to the fourfold intensification of drought impact on growing-season NEP_sat_ under future conditions (Fig. 3). Together, these findings highlight that extreme drought risk to C cycling will intensify with projected climate change (*45*). Conversely, in another grassland, which was unmanaged and received 40% less rainfall than ours, drought impact on net primary productivity was reduced by half under warmer, CO_2_-enriched conditions (*11*), suggesting that future drought resilience across grassland systems may be climate- and management-dependent.

### Implications

Our findings provide several key insights into ecosystem responses to multiple global change factors, demonstrating unexpectedly strong synergistic effects of eCO_2_, warming, and drought, especially as the number of coinciding factors increases (Fig. 2, fig. S3, and table S2). This suggests that the combined effects of multiple factors may play a greater role in shaping future ecosystem functioning than commonly assumed (*8*, *9*). Although future studies using a full-factorial structure could better partition the relative contributions of eCO_2_ and warming under drought, we expect such studies to corroborate the role of synergies in shaping ecosystem responses. In our study, hydroclimate emerged as a stronger mediator of global-change effects than the canopy structure (Figs. 4 and 5) despite the dynamics induced by mowing (Fig. 1). Therefore, more explicit incorporation of soil moisture-atmosphere feedbacks may improve the designs of future experiments aiming to disentangle the effects of global change drivers on ecosystem functioning (*59*–*61*). We note, however, that unmeasured factors, such as canopy greenness or species composition [e.g., (*62*, *63*)], could increase the apparent canopy influence (*48*). Nevertheless, many treatment effects are unlikely to be fully explained by hydroclimatic and canopy structure variables alone (Fig. 5). This points to sizable residual effects stemming from factor interactions, unidentified drivers, and cumulative preexposure to global change [e.g., (*64*)], all of which represent promising avenues for future research.

In conclusion, while eCO_2_ and warming have limited effects individually, their interaction synergistically amplifies R_eco_ and drought effects on key ecosystem functional properties (CO_2_ uptake, respiration, and carbon storage, as well as WUE and CUE). These synergistic effects can be influenced by altered soil processes and shifts in nonlinear ecosystem responses to declining moisture availability. From a carbon sink-source perspective, the enhanced strength and persistence of drought effects under warmer, CO_2_-enriched conditions are concerning, pointing to a declining ability of drought-exposed ecosystems to access water and achieve net C uptake as climate change advances.

## MATERIALS AND METHODS

### Experimental design

This study was conducted at a long-term multifactor global change experiment, “ClimGrass,” during the 2020 and 2021 growing seasons. This experiment is located in a managed grassland at the Raumberg-Gumpenstein research facility near the central European Alps in Styria, Austria (47°29′44.6″N, 14°5′54.6″E) (*32*, *39*, *43*, *47*, *48*, *50*, *51*). This site lies at 695 m above sea level, with a mean annual precipitation of 1077 mm and a mean annual temperature of 8.5°C, typical for lower montane Alpine valleys. The soil is a Cambisol (arenic and humic) with a loamy sand texture (sand: 44%; silt: 48%; clay: 8%). The vegetation is dominated by C_3_ grasses (*Arrhenatherum elatius* L., *Poa pratensis* L., *Festuca pratensis* L., and *Dactylis glomerata* L.) and forbs (*Taraxacum officinale*, *Trifolium repens*, and *Plantago lanceolata*), with canopy biomass composed of 84% grasses, 14% nonleguminous forbs, and 2% legumes (*48*). Grassland management follows traditional regional practice, with three annual mowing events timed to phenological development (late May, late July, and early October). Following each event, the nutrients removed during this biomass harvest are balanced by fertilization, which provides 90 kg N, 28 kg P, and 140 kg K per hectare annually. The growing seasons (April to October) were characterized by warm, wet conditions in 2020 and relatively average climatic conditions in 2021.

This study used 23 plots (4 by 4 m), which apply six global change treatments: (i) ambient conditions (control; *n* = 6), (ii) ambient drought conditions (*n* = 3), (iii) eCO_2_ (*n* = 3), (iv) warming (*n* = 3), (v) future conditions (eCO_2_ and warming; *n* = 4), and (vi) future drought conditions (eCO_2_, warming, and drought; *n* = 4) (*32*, *39*, *43*, *47*, *48*, *50*, *51*). The treatments of eCO_2_ and warming began in 2014 and use heaters and fumigation equipment mounted on aluminum frames suspended over plots, which are regularly adjusted to canopy height. The eCO_2_ treatment is applied using a mini free-air CO_2_ enrichment (“FACE”) system and aims to raise daytime CO_2_ concentrations, which averaged 420 ppm during sampling years, by 300 ppm. The warming treatment increases canopy surface temperatures by 3°C using heaters. The intensity of both eCO_2_ and warming treatments reflects a late-21st-century climate scenario, projected under “business as usual” emissions (*1*). In several ambient and future-climate plots, automatic shelters were added in 2017, which exclude mid-summer rainfall to induce annual drought treatments, suppressing precipitation by ~250 mm between approximately June and August (*39*). Drought treatments are ended with rewetting with 40 mm of collected rainwater, as well as a mowing event.

### Environmental data

Environmental data were collected throughout the growing seasons. VPD was calculated in each treatment using air temperature and relative humidity measured by permanently installed sensors (CS215-L, Campbell Scientific, Logan, UT). For chamber measurements, VPD was obtained using an HM75 sensor, which measured canopy-level air temperature and relative humidity inside the chamber (Vaisala, Helsinki, Finland). SWC (depth weighted from 0 to 25 cm) in the main rooting horizon was assessed using sensors at 3-, 9-, and 18-cm depths (Delta-T SM150, METER Group, Munich, Germany) connected to a datalogger (CR1000, Campbell Scientific, Logan, UT) (*32*, *39*). Rainfall data were obtained from an on-site weather station (GeoSphere Austria; www.geosphere.at).

### Ecosystem functional properties

Throughout the 2020 and 2021 growing seasons, closed-ecosystem chambers were used to take biweekly measurements of water and CO_2_ gas exchange [ET and net ecosystem exchange (NEE)], as well as R_eco_, following established protocols (*38*, *43*, *65*) and avoiding known pitfalls (*66*). Chambers (0.5 by 0.5 by 0.5 m; internally ventilated) were placed over preinstalled frames to enclose grassland subplots. Two types were used: transparent chambers for measuring ET and NEE under ambient light and opaque chambers for measuring R_eco_ in the darkness immediately afterward, isolating respiration from photosynthesis. For each plot, chamber fluxes were determined from minute-long changes in internal CO_2_ (GMP343, Vaisala, Helsinki, Finland) and gaseous H_2_O and temperature (HM75, Vaisala, Helsinki, Finland). Two criteria ensured that these measurements represent ecosystem functional properties [i.e., the ecosystem’s capacity to function (*2*, *3*)] over time. First, transparent-chamber measurements were taken under full-light conditions, with sampling restricted to sunny periods when the photon flux density exceeded 1400 μmol m^2^ s^−1^ (measured using an MQ-200 device; Apogee Instruments, Logan, US), associated with light-saturated GPP (indicated using subscript “sat”). Second, measurements were taken between 10:00 and 13:00 CET (central European time) when the photosynthetic activity is the highest, avoiding the afternoon when ET and R_eco_ may be inflated by high temperatures and VPD, and GPP is inhibited (*33*). FACE equipment was shifted for chamber access, so measurements reflect primarily the indirect effects of eCO_2_.

From flux measurements, we calculated ecosystem functional properties. First, NEE_sat_ was converted to light-saturated NEP (NEP_sat_)—the rate at which CO_2_ is accumulated (or lost) in the system

$${\text{NEP}}_{\text{sat}}=-1\times {\text{NEE}}_{\text{sat}}$$ (1)

Positive NEP_sat_ reflects the system’s capacity to accumulate CO_2_, whereas negative values indicate that the system is acting as a CO_2_ source. Then, combining NEE_sat_ with the coupled R_eco_ measurement, we inferred the gross light-saturated uptake of CO_2_ (GPP_sat_)

$${\text{GPP}}_{\text{sat}}={\text{NEP}}_{\text{sat}}+R_{\text{eco}}$$ (2)

This approach has two known limitations. First, it assumes a closed CO_2_ balance, although time lags between photosynthetic uptake and subsequent respiration can cause small over- or underestimates of GPP_sat_. Aggregating measurements over longer periods, as done in our study, helps smooth out these short-term mismatches because respiratory fluxes arising from earlier assimilation become distributed across the averaging window, reducing sensitivity to momentary mismatches compared to single measurements. Second, leaf respiration may be partly suppressed under full light, whereas R_eco_ was measured in the darkness, which can introduce independent discrepancies between R_eco_ and NEE_sat_ (*66*), although these should be minor in our study as most of the grassland’s respiration occurs in the soil (*51*). Building on GPP_sat_, we calculated CUE_sat_, representing the maximum proportion of C retained after respiration by grassland on sampling days, as

$$\text{CUE}=1-\frac{R_{\text{eco}}}{{\text{GPP}}_{\text{sat}}}$$ (3)

Last, we calculated underlying WUE_sat_—reflecting the maximum ecosystem CO_2_ uptake per unit water use on each sampling day—while accounting for subdaily variation in VPD, as

$$\text{WUE}=\frac{{\text{GPP}}_{\text{sat}}\sqrt{\text{VPD}}}{{\text{ET}}_{\text{sat}}}$$ (4)

as described in a previous study (*33*). Notably, although not included in this study as an ecosystem functional property, we also investigated grassland canopy conductance (fig. S8) as per previous studies (*32*, *39*), measured as chamber-derived ET_sat_/VPD.

### Structural properties

To assess how the canopy structure influenced ecosystem functional properties, we recorded canopy height and LAI throughout the field seasons. Canopy height, which reflects the vertical structure and growth potential of the grassland, was measured biweekly using a ruler. LAI, which quantifies the leaf surface area for light interception and gas exchange, was measured weekly using an AccuPAR LP-80 (METER Group, Munich, Germany). This device calculates LAI on the basis of canopy light transmittance, accounting for solar angle, beam fraction, and leaf angle distribution. Because measurements in our study were taken between 11:00 and 13:00 CET on sunny days, no correction was needed for leaf angle distribution—the only parameter potentially sensitive to treatments. AccuPAR LP-80 measurements closely matched manual LAI estimates between 0.5 and 5 in a nearby grassland (*67*). To ensure data reliability, we excluded values below this range from statistical analyses. Values above 5 were rare and had no significant impact on our results.

### Statistical analysis

All analyses were performed in R software version 4.3.3 (*68*). First, we used linear mixed-effect models using the “lme4” package (*69*), both to compare ecosystem functional properties across treatments (*n* = 6) and to test for nonadditive interactions within 2 × 2 factorial designs (where *n* = 4 treatments). While we adopt a conventional significance threshold (α = 0.05) for identifying treatment effects, we also report more limited evidence (α = 0.1) to support the interpretation of coordinated ecosystem responses. Models were run separately for each property, with random intercept effects for date and plot to ensure that estimated treatment effects were consistent in time and space (*32*). For the comparison of properties across six treatments, we used a model specified as “lmer (ecosystem functional property ~ treatment + (1|date) + (1|plot), data = data).” Marginal effects of treatments were estimated using the “emmeans” function [“emmeans” package (*70*)] combined with a false discovery rate correction from the “cld” function [“multcomp” package (*71*)]. Across these models, plot-intercept variance was minor (<12% of residual variance; table S1), indicating that plots act as reliable treatment replicates (*43*). Then, for the testing of additive versus nonadditive effects, we made use of three available factorial designs: (i) warming_yes/no_ × eCO_2 yes/no_, (ii) drought_yes/no_ × future climate_yes/no_, and (iii) postdrought_yes/no_ × future climate_yes/no_. Each factorial design was tested separately using a model formulated as “lmer (ecosystem functional property ~ factor_1_ × factor_2_ + (1|date) + (1|plot), data = data).” Main effects and interactions were evaluated using type III Wald chi-square tests [“car” package (*72*)]. For all models, we verified that the assumptions of normality and homoscedasticity were not violated. Model fit was evaluated using marginal and conditional *R*^2^ values (*R*^2^*m* and *R*^2^*c*) (*73*).

In a next step, we aimed to identify the marginal nonlinear effects of multiple potential drivers while effectively handling correlated predictors and interactions between effects. To achieve this, we used random forest regression with 500 trees and default settings for each functional property [“randomForest” package (*74*)]. Predictors included canopy structure (height and LAI), hydroclimatic conditions (temperature, VPD, and SWC), time (month and year), and global change treatment. The inclusion of treatment allowed the estimation of a “residual” effect, reflecting the portion of the total treatment effect not explained by other predictors. Model performance was assessed using out-of-bag *R*^2^ values (*75*). To ensure the robustness of our results given our modest sample size, we performed a sensitivity analysis by reducing model complexity (by increasing node size, i.e., requiring each tree decision to be based on at least 20 observations rather than the default 5). This adjustment reduced *R*^2^ across models by a maximum of only 0.02 (table S3), indicating that identified drivers are robust to model simplification and do not depend on highly specific tree decisions and that default models are therefore unlikely to be overfit to noise. The relative importance of predictors was computed by permuting out-of-bag data [“importance” function (*74*)]. For each tree, the out-of-bag portion of the data was calculated as the mean square error. Then, the same was done after permuting each predictor variable. The difference between the two was then averaged over all trees and normalized by the standard deviation of the differences, yielding the “% increase in mean square error.”

To interpret random forest model predictions, we calculated SHAP values [“shapviz” package (*76*)], which quantify the marginal contribution of each predictor to the model (*77*). To estimate uncertainty around these SHAP values, we repeated this analysis across 100 bootstrapped datasets, generated by randomly resampling the original data with replacement. The standard deviation of SHAP values across bootstraps can be used to estimate the random error (*78*–*80*) and is shown as a range around marginal predictor contributions (fig. S5).

Last, to assess how different predictors underpinned treatment effects, we predicted synthetic outcomes with random forest models in which treatment-induced variation in predictors was removed by substituting ambient values. Although random forest approaches are relatively robust to multicollinearity, predictors were grouped by type (canopy, hydroclimate, or both) to avoid overinterpreting individual variables, some of which were correlated (e.g., temperature and VPD). We then compared how each substitution altered estimated treatment effects, quantifying the relative contribution of each group. Notably, this analysis does not account for interactions between predictor groups, which may be partially reflected by the residual effect, slightly inflating its apparent magnitude.
