# Supplementary material for: Synergistic effects of warming and elevated CO2 intensify drought impacts on grassland carbon and water fluxes
Source: Sci Adv. 2026 Jun 26;12(26):eaea8988. doi: 10.1126/sciadv.aea8988 (PMC13308592; doi:10.1126/sciadv.aea8988)
Supplement: Supplementary file 1 — Figs. S1 to S9 Tables S1 to S3 [file sciadv.aea8988_sm.pdf]

Science Advances
AAAS

Supplementary Materials for

Synergistic effects of warming and elevated CO_2 intensify drought impacts on grassland carbon and water fluxes

Maud Tissink *et al.*

Corresponding author: Maud Tissink, maud.tissink@uibk.ac.at; Michael Bahn, michael.bahn@uibk.ac.at

Sci. Adv. **12**, eaea8988 (2026)
DOI: 10.1126/sciadv.aea8988

This PDF file includes:

Figs. S1 to S9
Tables S1 to S3

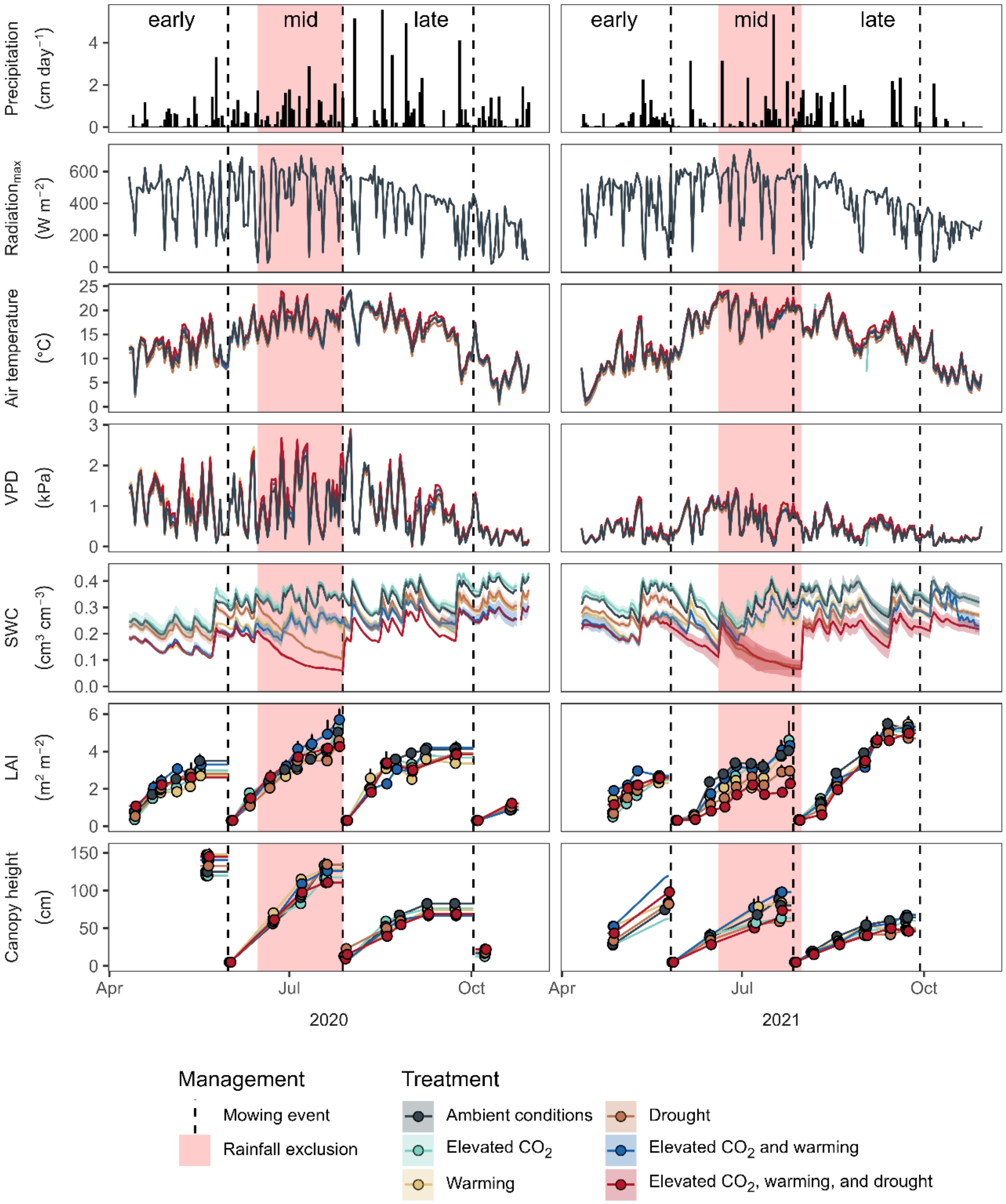

Fig. S1. Growing-season conditions across global change treatments—extended. Daily precipitation, maximum daily radiation, air temperature, vapor pressure deficit (VPD), soil water content (SWC, depth-weighted from 0 to 25 cm), leaf area index (LAI), and canopy height in a grassland experiment over two growing seasons. Mowing events occurred three times per year, defining the early, mid, and late periods of each growing season. Treatments include individual and combined applications of elevated CO_2 (+300 ppm), warming (+3°C canopy surface temperature) and drought (summer rainfall exclusion, ended with a 40 mm rewetting). Coloured lines indicate mean treatment values over time, with ribbons denoting standard errors (gap-filled). Points show treatment means for individual sampling campaigns ($N = 3$ per plot; see Methods for the number of plots per treatment); here, standard errors are represented using error bars.

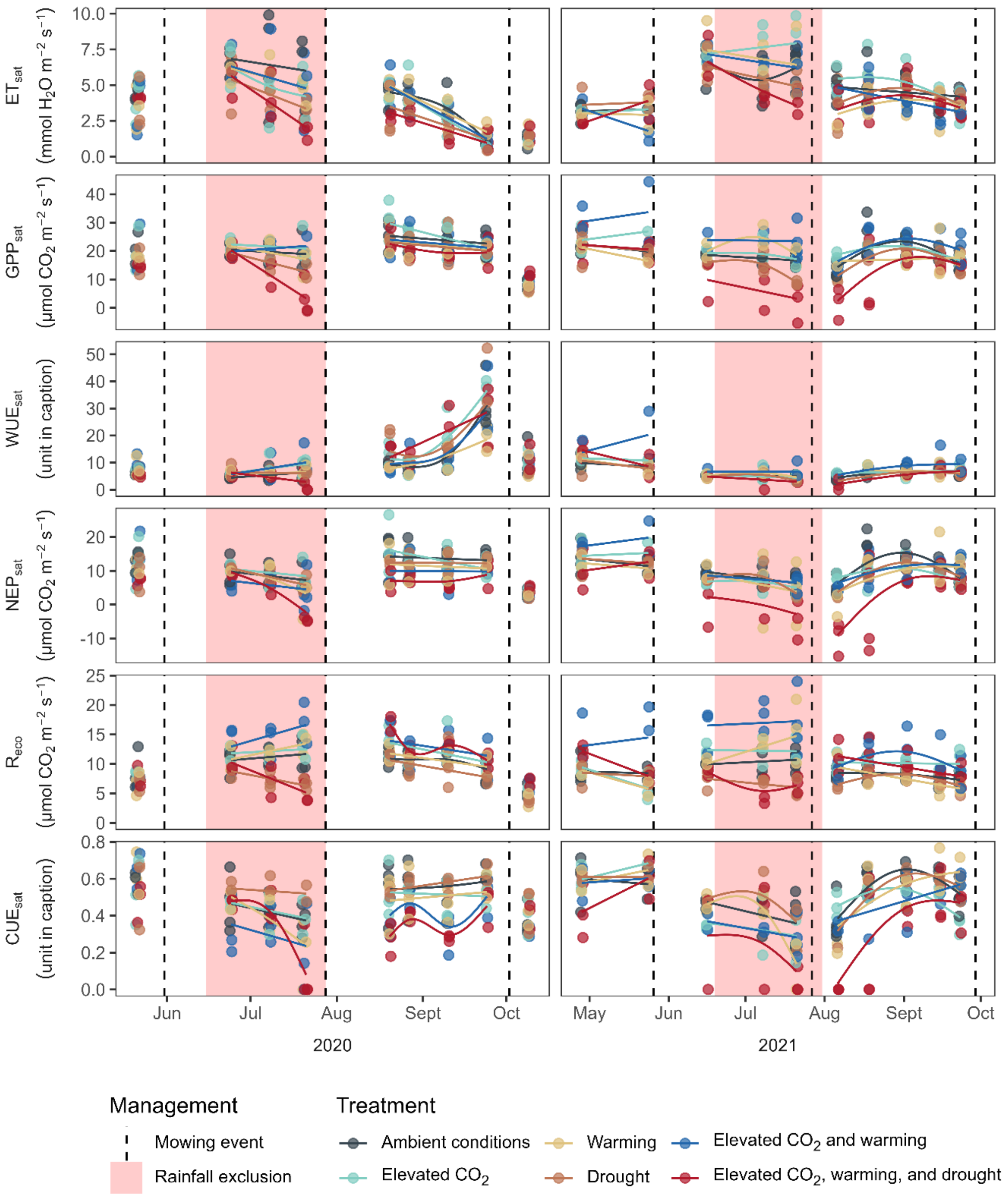

Fig. S2. Effects of individual and combined treatments of elevated CO_2, warming and drought on ecosystem functional properties related to water and CO_2 fluxes over time. Dots represent replicate samples across treatment over two growing seasons, with lines reflecting trends. Abbreviations: net ecosystem productivity (NEP), gross primary productivity (GPP), ecosystem respiration (R_{eco}), evapotranspiration (ET), carbon-use efficiency (CUE, expressed as µmol CO_2 NEP µmol^{-1} CO_2 GPP) and water-use efficiency (WUE, expressed as µmol CO_2 kPa$^{-0.5}$ mmol^{-1} H_2O). The subscript '$_{sat}$' indicates ecosystem functional properties associated with light-saturated GPP.

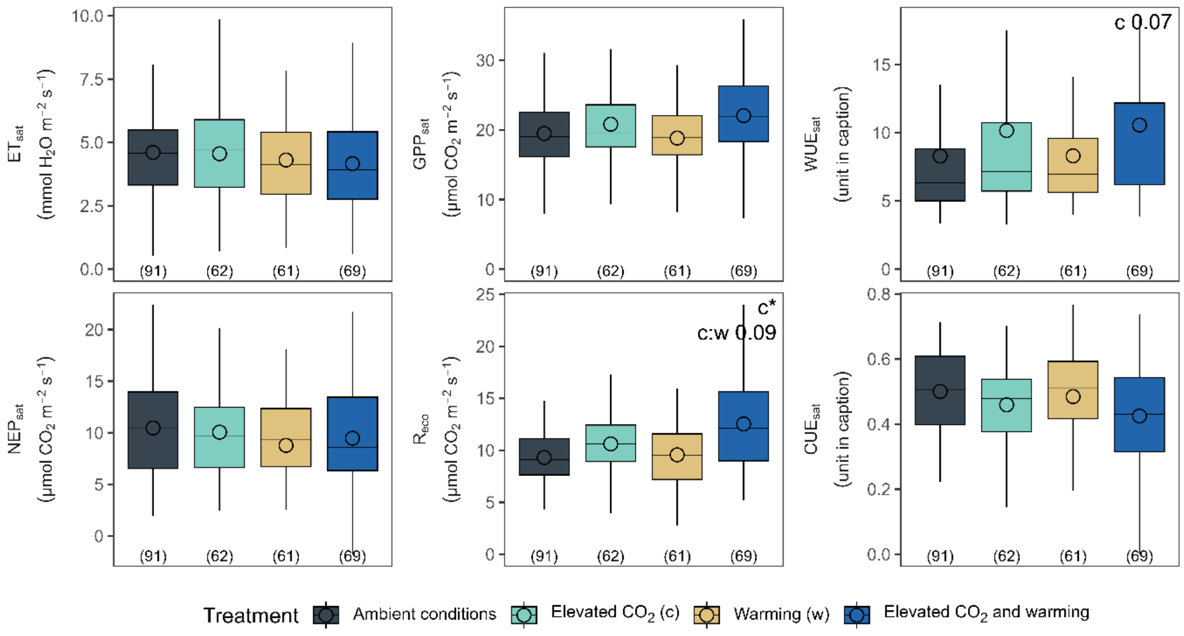

Fig. S3. Ecosystem functional properties related to water and CO_2 fluxes across individual and combined treatments of elevated CO_2 and warming.

Lower and upper boundaries of the boxplots indicate the 25th and 75th quartiles of replicate samples across treatments over two growing seasons, center lines indicate median values, and whiskers indicate 1.5 times the interquartile range (*N* is reported in brackets below each box; source data: **Fig. S2**). Points inside boxes are estimated means from linear mixed models with treatments as fixed effects, and random intercept effects for plot and date. Text indicates treatments and non-additive interactions with a statistically significant effect (abbreviations in figure legend; detailed statistics: **Table S2**). Abbreviations: net ecosystem productivity (NEP), gross primary productivity (GPP), ecosystem respiration (R_{eco}), evapotranspiration (ET), carbon-use efficiency (CUE, expressed as µmol CO_2 NEP μmol^{-1} CO_2 GPP) and water-use efficiency (WUE, expressed as µmol CO_2 $kPa^{-0.5}$ $mmol^{-1}$ H_2O). The subscript '$_{sat}$' indicates ecosystem functional properties associated with light-saturated GPP.

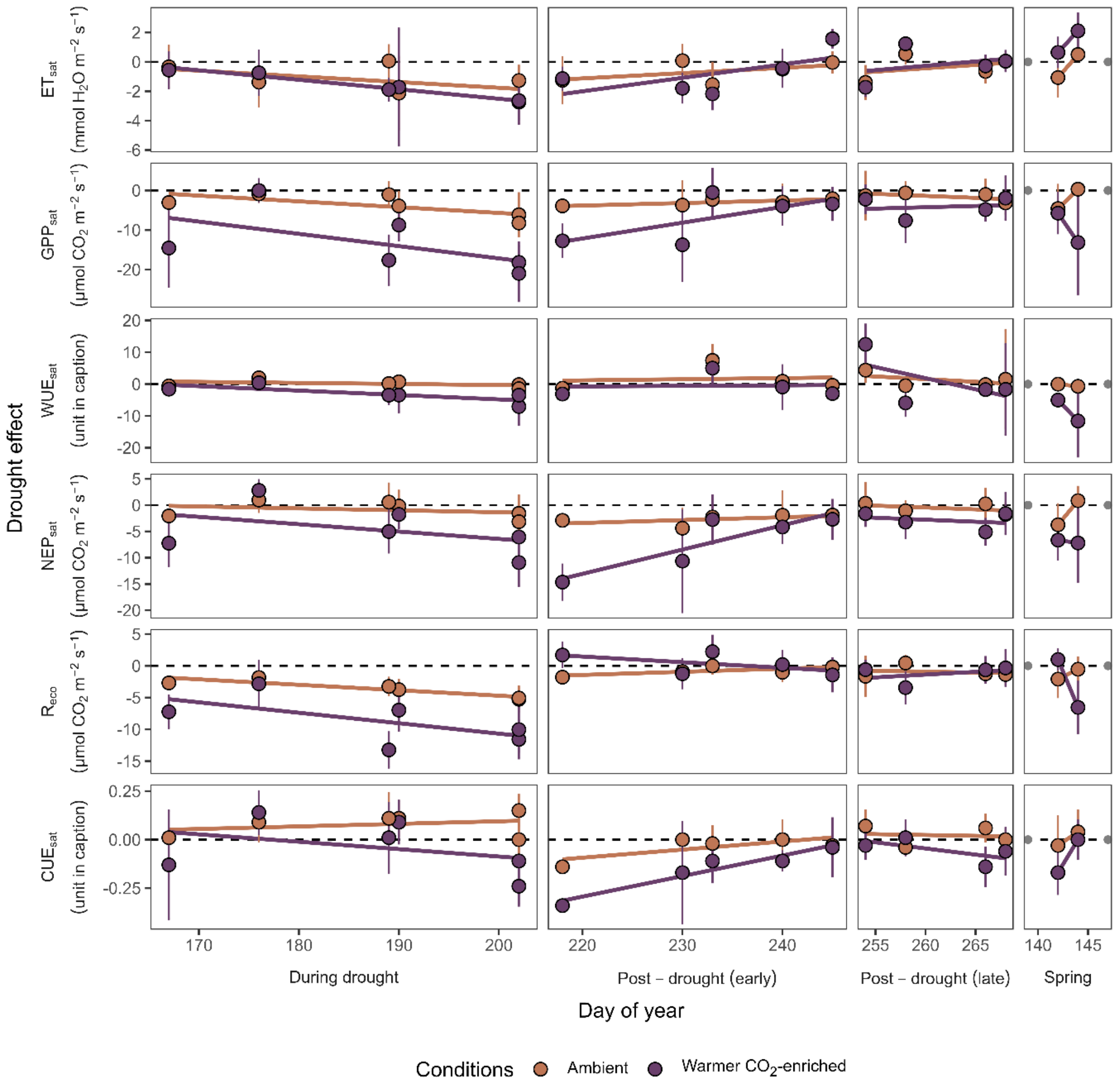

Fig. S4. Drought effects on ecosystem functional properties related to water and CO_2 fluxes under ambient versus warmer, CO_2-enriched conditions.

Drought effects are compared over four distinct periods: the drought events themselves, the early post-drought phase (first three weeks after drought), the late post-drought phase (from week four after drought until snowfall), and the following spring. Points represent treatment means (see Methods for the number of plots per treatment), error bars denote standard errors, and lines reflect trends. Abbreviations: net ecosystem productivity (NEP), gross primary productivity (GPP), ecosystem respiration (R_{eco}), evapotranspiration (ET), carbon-use efficiency (CUE, expressed as µmol CO_2 NEP µmol^{-1} CO_2 GPP) and water-use efficiency (WUE, expressed as µmol CO_2 kPa$^{-0.5}$ mmol^{-1} H_2O). The subscript '$_{sat}$' indicates ecosystem functional properties associated with light-saturated GPP.

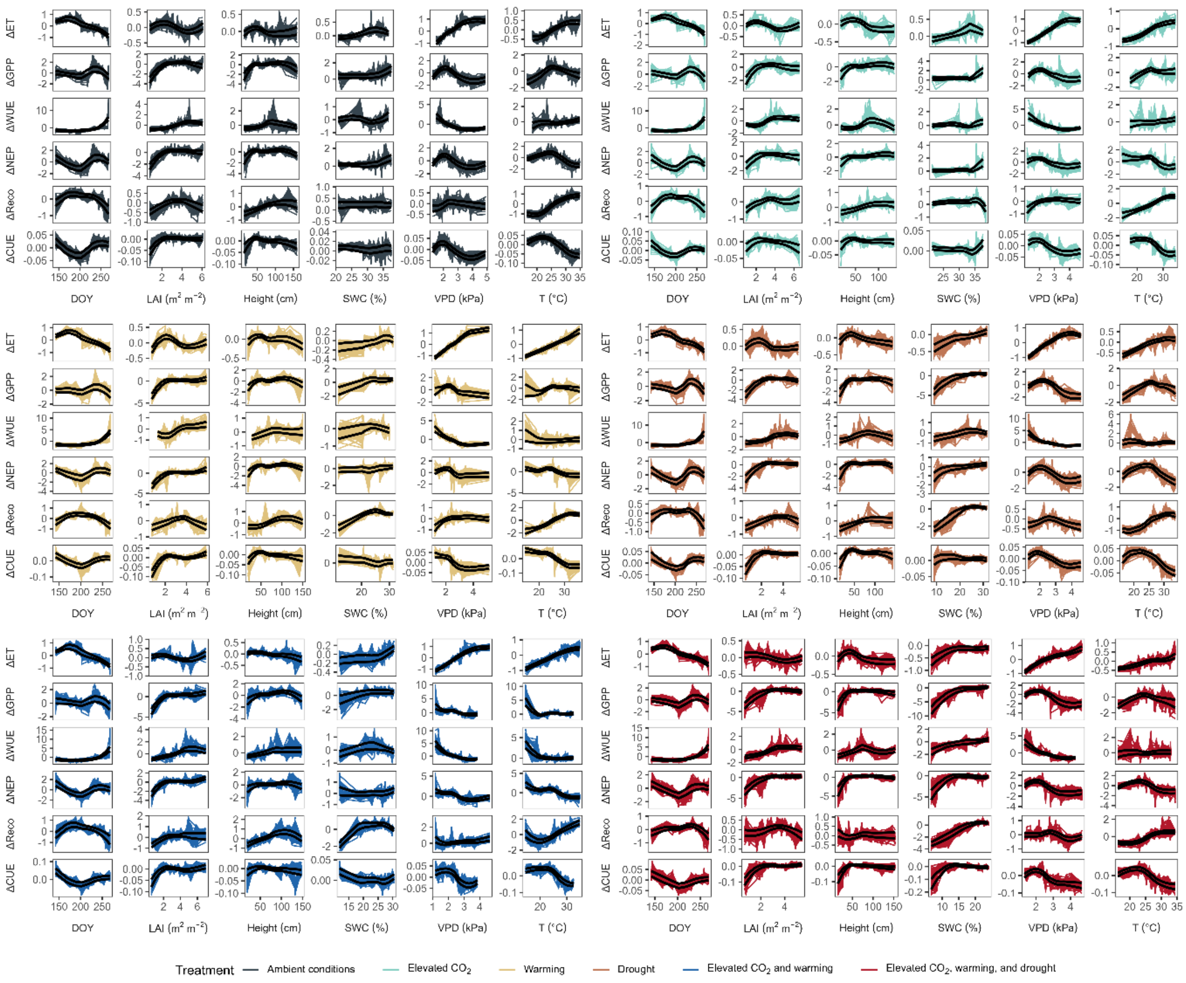

Fig. S5. Shapley Additive exPlanations (SHAP) values derived from 100 bootstrapped datasets.

Shown are the marginal contributions of predictors to variation in ecosystem functional properties related to water and CO_2 fluxes across six global change treatments, predicted by Random Forest regression models. Black lines (LOESS models) show standard deviations around the average model output and indicate likely error ranges, used for treatment comparisons (full details: **Figs. 4, S6**).

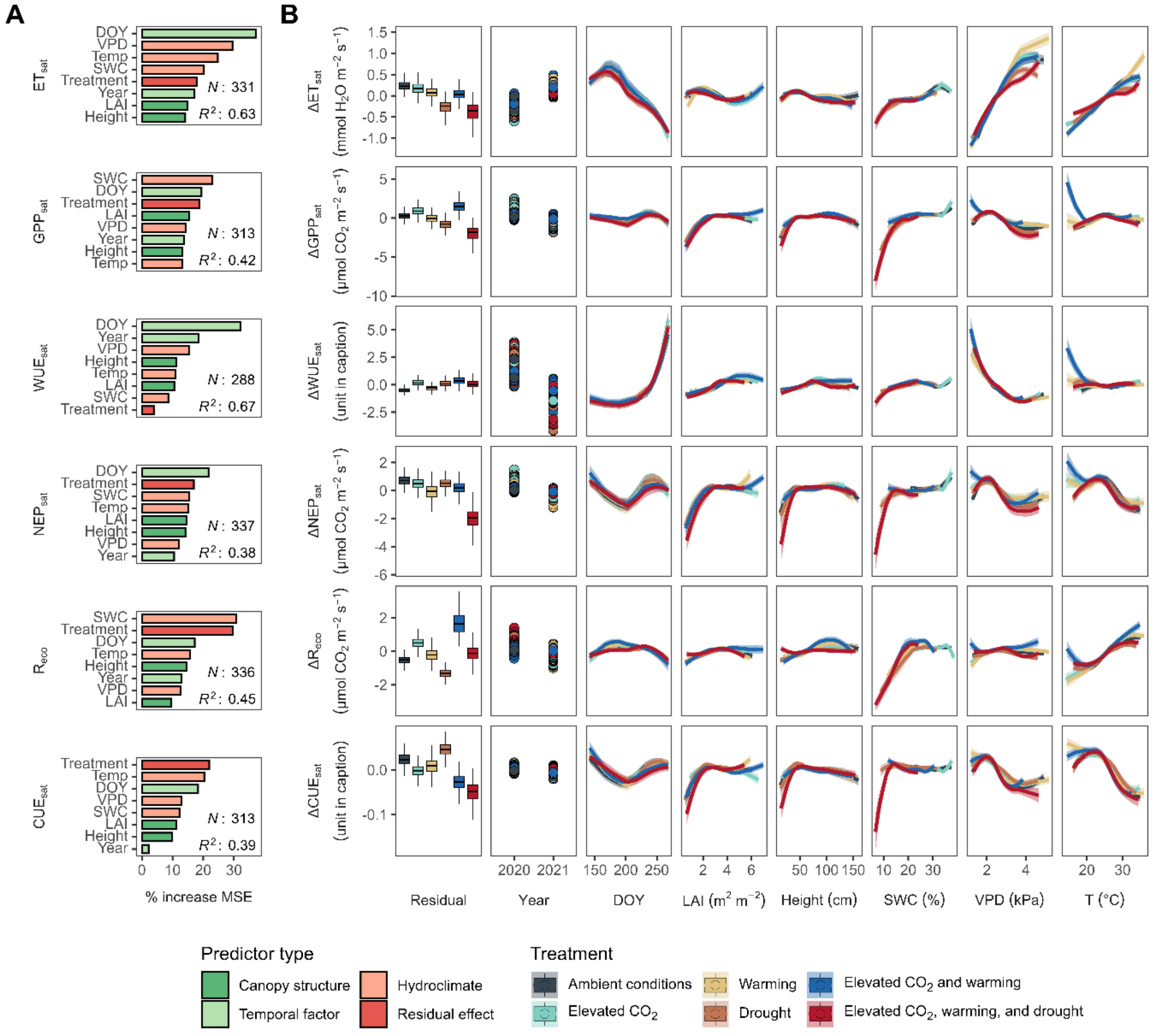

Fig. S6. Importance and contributions of predictors to ecosystem functional properties related to water and CO_2 fluxes under global change treatments. (A) Relative predictor importance of each property ("% increase in MSE", where MSE is mean square error) as determined using random forest models (*N* and R^2 values reported). **(B)** Marginal predictor contributions to each property, as indicated by Shapley Additive exPlanations (SHAP) values. 'Residual' effects represent any portion of the treatment effect not explained by predictors alone. Shaded areas indicate error ranges, calculated as standard deviations from 100 bootstrapped datasets (**Fig. S5**). Abbreviations: net ecosystem productivity (NEP), gross primary productivity (GPP), ecosystem respiration (R_{eco}), evapotranspiration (ET), carbon-use efficiency (CUE, expressed as μmol CO_2 NEP μmol^{-1} CO_2 GPP) and water-use efficiency (WUE, expressed as μmol CO_2 kPa$^{-0.5}$ mmol^{-1} H_2O). The subscript '$_{sat}$' indicates ecosystem functional properties associated with light-saturated GPP. Predictor abbreviations: temperature (T/Temp), soil water content (SWC), vapor pressure deficit (VPD), leaf area index (LAI), and day of year (DOY).

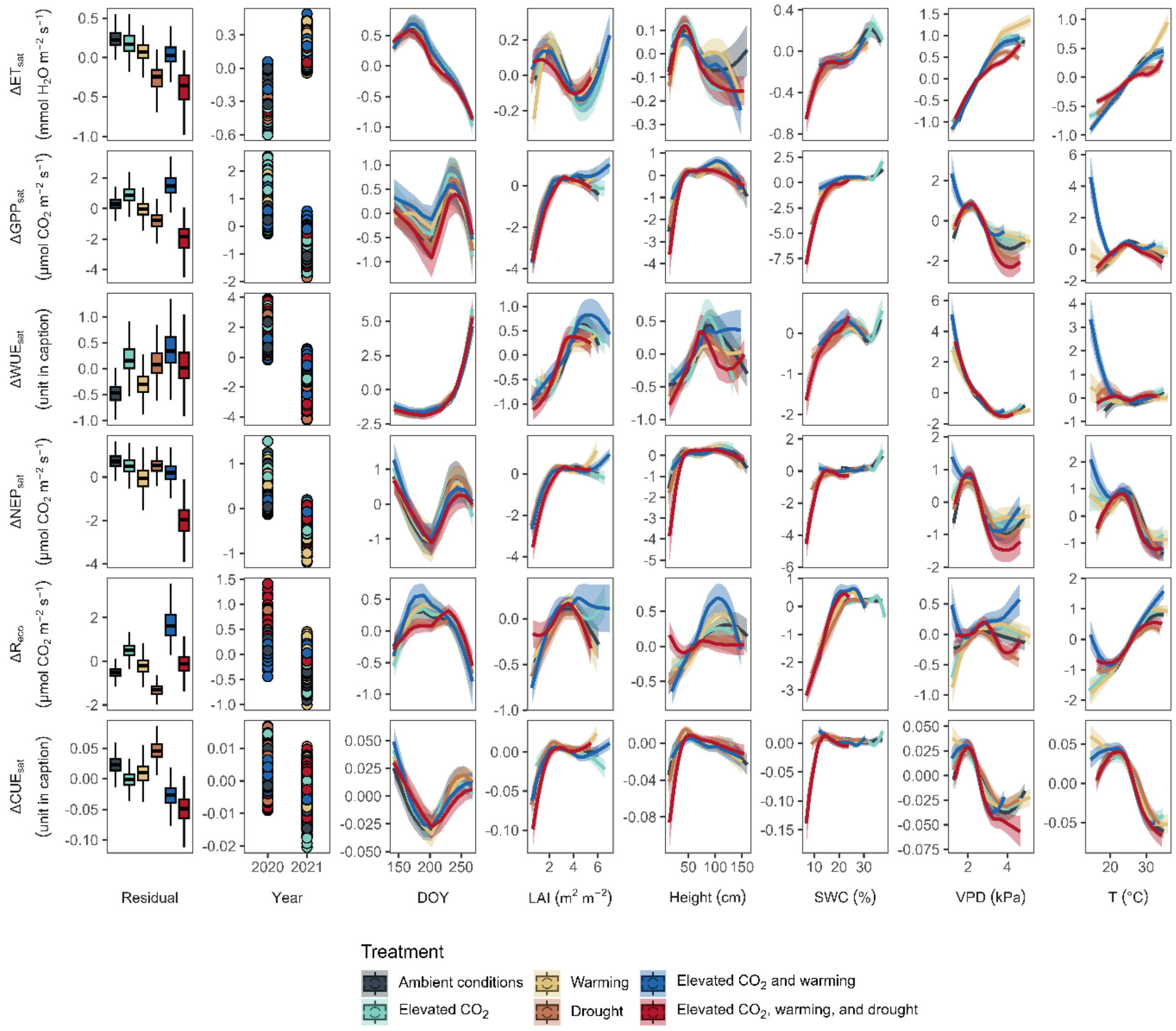

Fig. S7. Representation of Figures 4B and S6B with independent facet axes for clearer comparison of treatment effects.

(full details: **Figs. 4B, S6B**).

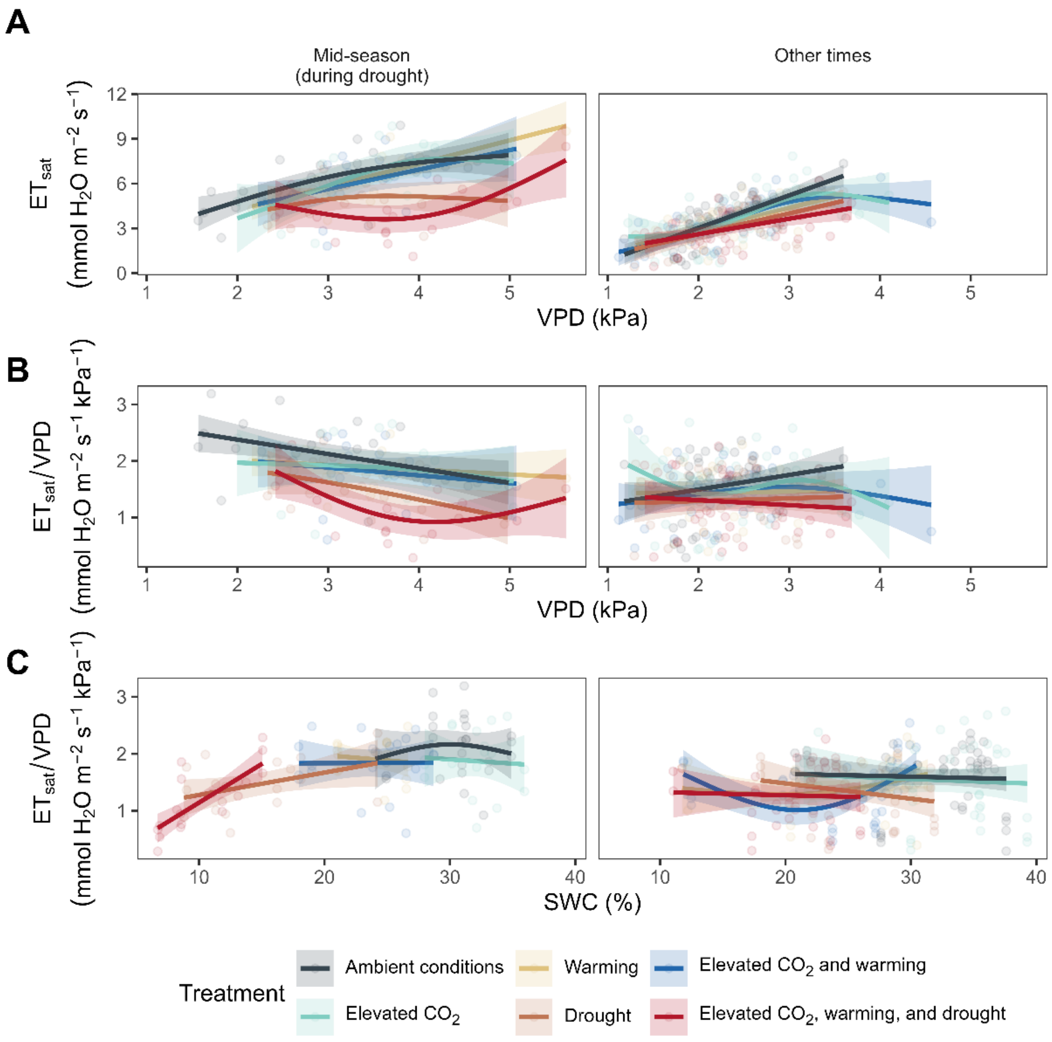

Fig. S8. Ecosystem water-use relative to soil and atmospheric water availability. Dots represent replicate samples across treatment over two growing seasons, lines reflect trends (LOESS models), and ribbons show 95% confidence intervals. Note that values for water use (ET_{sat}) are associated with light-saturated gross primary productivity. **(A)** Responses of ET_{sat} to vapor pressure deficit (VPD) across six global change treatments. **(B)** Responses of ET_{sat} normalized by VPD (ET_{sat}/VPD), defined here as canopy conductance, to VPD. **(C)** Responses of canopy conductance to soil water content (SWC).

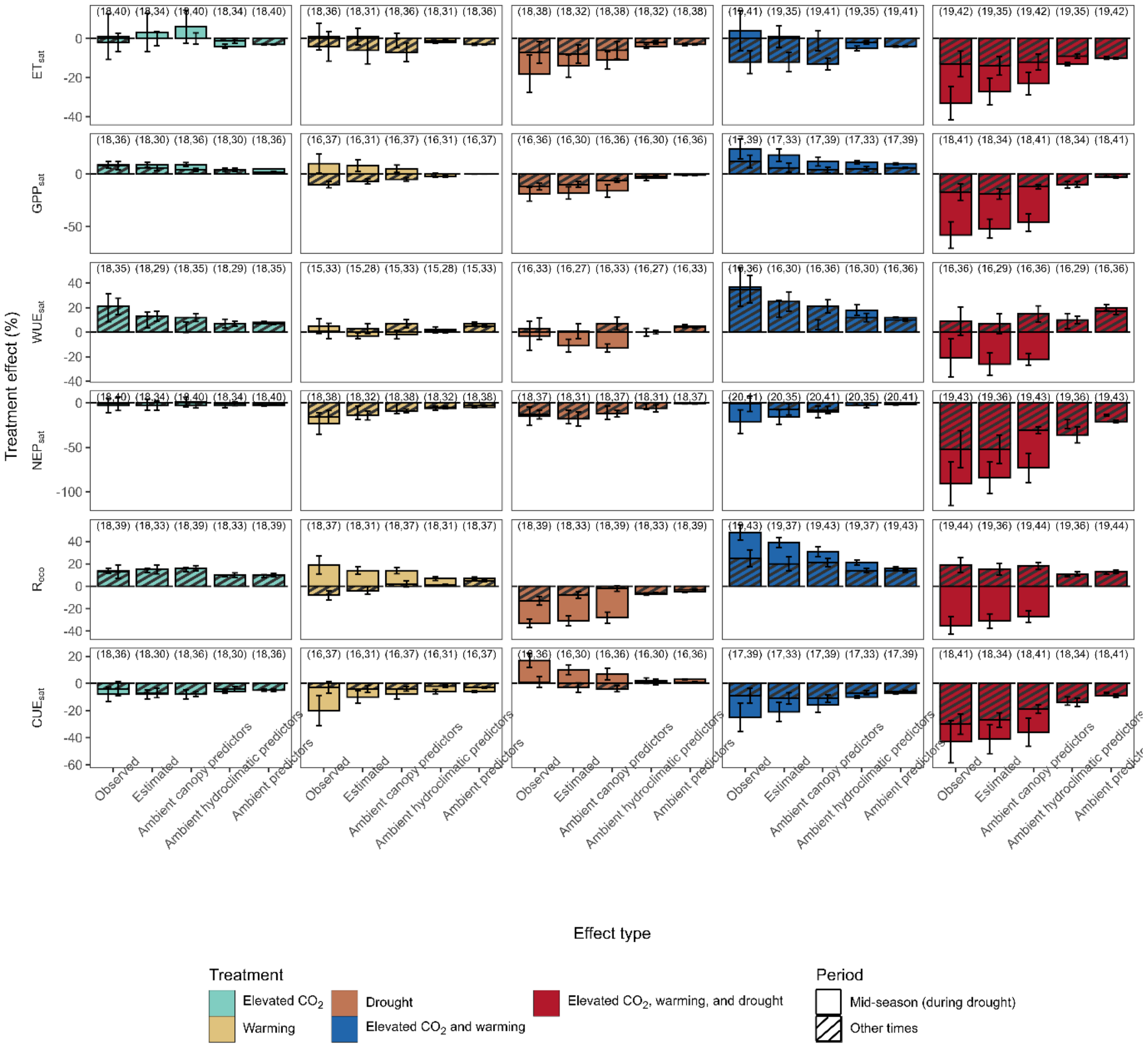

Fig. S9. Observed and estimated effects of individual and combined treatments of elevated CO_2, warming and drought on ecosystem functional properties related to water and CO_2 fluxes.

Shown are observed and model-estimated effects on properties during mid-season periods and at other times. Estimated treatment effects ("Estimated", "Ambient canopy predictors", "Ambient hydroclimatic predictors", and "Ambient predictors", where the final estimate type is based on ambient imputed values for both canopy and hydroclimatic predictors) reveal the modelled contribution of canopy structure and hydroclimate conditions to global change effects across treatments. Labels report sample sizes, presented as "($N_{\text{mid-season}}$, $N_{\text{other times}}$)". Abbreviations: net ecosystem productivity (NEP), gross primary productivity (GPP), ecosystem respiration (R_{eco}), evapotranspiration (ET), carbon-use efficiency (CUE) and water-use efficiency (WUE). The subscript '$_{sat}$' indicates ecosystem functional properties associated with light-saturated GPP.

Table S1. Effects of global change factors on ecosystem functional properties related to water and CO_2 fluxes.

Results of type III Wald chi-square tests on linear mixed-effect models with treatment as a fixed effect (Df = 5), and random intercept effects for plot and date (reported as variances; σ^2). Fixed effects from linear models are reported as estimated means ± standard error (SE), followed in brackets by a percentage change relative to ambient conditions including a 95% confidence interval (CI). The statistical significance for the treatment Chi-square statistic (χ^2) is reported using asterisks (***P < 0.001; **P < 0.01; *P < 0.05). Further abbreviations represent the sample size (N), marginal R^2 (R^2m) and conditional R^2 (R^2c) of the models.

Model parameters								Estimated fixed effects					
Property	N	R^2m	R^2c	σ^2 *plot*	σ^2 *date*	σ^2 *residual*	χ^2	*Ambient conditions*	*Ambient drought*	*Warming*	*Elevated CO_2*	*Future conditions*	*Future drought*
ET_{sat}	369	0.04	0.6	0	2.02	1.52	**35.9*****	4.45 ± 0.35	−0.68 ± 0.22 (−15.3% [−25.1, −5.5])	−0.22 ± 0.22 (−4.9% [−14.8, +5.0])	0.06 ± 0.21 (+1.3% [−8.0, +10.7])	−0.33 ± 0.21 (−7.4% [−16.7, +1.9])	−1.03 ± 0.21 (−23.1% [−32.4, −13.8])
GPP_{sat}	352	0.16	0.58	1.6	14.88	19.45	**37.5*****	19.5 ± 1.26	−2.78 ± 1.49 (−14.3% [−29.3, +0.7])	−0.97 ± 1.49 (−5.0% [−20.0, +10.0])	1.37 ± 1.48 (+7.0% [−7.9, +21.8])	2.25 ± 1.42 (+11.5% [−2.7, +25.7])	−5.97 ± 1.40 (−30.6% [−44.6, −16.5])
WUE_{sat}	326	0.02	0.66	0.13	29.92	16.9	10.2	8.33 ± 1.36	0.50 ± 0.94 (+6.0% [−16.0, +28.0])	−0.80 ± 0.95 (−9.6% [−32.0, +12.8])	1.62 ± 0.92 (+19.4% [−2.3, +41.0])	1.89 ± 0.91 (+22.7% [+1.3, +44.1])	0.43 ± 0.92 (+5.2% [−16.5, +26.8])
NEP_{sat}	376	0.14	0.57	1.66	11.09	14.86	**29.1*****	10.6 ± 1.13	−1.53 ± 1.40 (−14.4% [−40.4, +11.6])	−1.82 ± 1.40 (−17.2% [−43.2, +8.8])	−0.63 ± 1.39 (−5.9% [−31.7, +19.9])	−1.31 ± 1.32 (−12.4% [−36.8, +12.0])	−6.68 ± 1.32 (−63.0% [−87.5, −38.5])
R_{eco}	377	0.18	0.51	0.18	3.05	5.81	**60.7*****	9.16 ± 0.56	−1.68 ± 0.63 (−18.3% [−31.8, −4.9])	0.31 ± 0.63 (+3.4% [−10.1, +16.9])	1.39 ± 0.63 (+15.2% [+1.7, +28.7])	3.29 ± 0.60 (+35.9% [+23.0, +48.8])	0.35 ± 0.60 (+3.8% [−9.1, +16.8])
CUE_{sat}	352	0.14	0.57	0	0.01	0.01	**45.7*****	0.51 ± 0.03	0.02 ± 0.03 (+3.9% [−7.7, +15.5])	−0.03 ± 0.03 (−5.9% [−17.5, +5.7])	−0.05 ± 0.03 (−9.8% [−21.4, +1.8])	−0.09 ± 0.03 (−17.6% [−29.2, −6.0])	−0.18 ± 0.03 (−35.3% [−46.9, −23.7])

Table S2. Individual versus combined effects of factorial global change on ecosystem functional properties related to water and CO_2 fluxes.

Results of type III Wald chi-square tests on linear mixed-effect models with treatment factorials as fixed effects, and random intercept effects for plot and date (reported as variances; σ^2). Fixed effects from linear models are reported as estimated means ± standard error (SE), followed in brackets by a percentage change relative to ambient conditions including a 95% confidence interval (CI). Three full factorial designs were tested. The statistical significance for the treatment Chi-square statistic (χ^2) is reported using asterisks (****P* < 0.001; ***P* < 0.01; **P* < 0.05). Further abbreviations represent the sample size (*N*), marginal R^2 (R^2m) and conditional R^2 (R^2c) of the models.

**Model parameters**								**Estimated fixed effects**						
Property	Factorial design	***N***	R^2m	R^2c	σ^2 *plot*	σ^2 *date*	σ^2 *residual*	Intercept *(ambient conditions)*	Treatment effect 1 *(warming / drought)*		Treatment effect 2 *(elevated CO_2 / warming and elevated CO_2)*		Interaction effect *(Treat_1 x Treat_2)*	
								β ± SE (i.e., the control value)	*β* ± SE (% change relative to control [95% CI])	χ^2	*β* ± SE (% change relative to control [95% CI])	χ^2	*β* ± SE (% change relative to control [95% CI])	χ^2
ET_{sat}	warming x elevated CO_2	252	0.01	0.63	0	2.47	1.57	4.44 ± 0.38	-0.21 ± 0.24 (−4.7% [−15.3, 5.9])	0.8	0.08 ± 0.23 (1.8% [−8.4, 12.0])	0.1	-0.18 ± 0.34 (−7.0% [−14.0, 28.0])	0.3
	drought x future conditions	82	0.17	0.38	0	0.63	2.43	6.07 ± 0.47	-1.26 ± 0.51 (−20.8% [−37.2, −4.3])	6.2*	-0.11 ± 0.50 (−1.8% [−18.0, 14.3])	0	-0.55 ± 0.73 (−31.6% [−1.4, 64.6])	0.6
	post-drought x future conditions	175	0.03	0.55	0	1.02	0.94	3.71 ± 0.31	-0.44 ± 0.21 (−11.9% [−23.0, −0.8])	4.4*	-0.46 ± 0.20 (−12.4% [−23.0, −1.8])	5.1*	0.22 ± 0.30 (−18.3% [−40.3, 3.7])	0.5
GPP_{sat}	warming x elevated CO_2	241	0.04	0.55	1.4	13.36	15.46	19.62 ± 1.18	-0.82 ± 1.35 (−4.2% [−11.0, 2.6])	0.4	1.44 ± 1.35 (7.3% [−6.1, 20.8])	1.1	1.66 ± 2.00 (11.6% [−16.0, 39.2])	0.7
	drought x future conditions	75	0.46	0.63	1.87	2.85	18.47	18.35 ± 1.54	-3.85 ± 2.13 (−21.0% [−33.6, −8.5])	3.3	3.53 ± 2.05 (19.2% [−2.7, 41.1])	3	-9.28 ± 3.10 (−52.3% [−98.1, −6.5])	9**
	post-drought x future conditions	170	0.1	0.64	3.2	19.99	17.99	20.15 ± 1.69	-2.41 ± 1.91 (−12.0% [−21.5, −2.6])	1.6	1.58 ± 1.80 (7.8% [−1.4, 16.9])	0.8	-3.58 ± 2.73 (−21.9% [−58.7, 14.9])	1.7
WUE_{sat}	warming x elevated CO_2	225	0.03	0.67	0.18	26.15	14.34	8.48 ± 1.29	-0.72 ± 0.93 (−8.5% [−19.5, 2.4])	0.6	1.65 ± 0.90 (19.5% [−1.1, 40.1])	3.4	0.95 ± 1.35 (22.2% [−21.0, 65.4])	0.5
	drought x future conditions	72	0.16	0.18	0	0	5.75	5.23 ± 0.51	0.16 ± 0.79 (3.1% [−12.3, 18.5])	0	2.00 ± 0.79 (38.3% [21.7, 54.9])	6.4*	-3.28 ± 1.18 (−21.4% [−82.3, 39.5])	7.8**
	post-drought x future conditions	153	0.01	0.66	1	40.04	23.31	9.82 ± 2.01	0.59 ± 1.62 (6.0% [−9.9, 21.9])	0.1	1.77 ± 1.55 (18.0% [−6.3, 42.4])	1.3	-1.29 ± 2.32 (−10.9% [−53.5, 75.3])	0.3
NEP_{sat}	warming x elevated CO_2	259	0.02	0.49	1.02	9.7	13.68	10.53 ± 1.02	-1.70 ± 1.19 (−16.1% [−27.6, −4.6])	2.1	-0.54 ± 1.18 (−5.1% [−16.3, 6.1])	0.2	1.01 ± 1.75 (−11.7% [−56.8, 33.4])	0.3
	drought x future conditions	83	0.25	0.6	2.01	3.42	9.49	7.75 ± 1.34	-0.76 ± 1.75 (−9.8% [−32.0, 12.4])	0.2	-1.33 ± 1.67 (−17.2% [−39.1, 4.6])	0.6	-4.11 ± 2.55 (−80.0% [−168.9, 8.9])	2.6
	post-drought x future conditions	179	0.17	0.59	2.08	10.96	15.64	11.9 ± 1.34	-1.83 ± 1.62 (−15.4% [−28.9, −1.9])	1.3	-1.19 ± 1.53 (−10.0% [−22.8, 2.7])	0.6	-3.50 ± 2.31 (−54.8% [−107.7, −1.9])	2.3
R_{eco}	warming x elevated CO_2	256	0.14	0.59	0.23	4.62	5.02	9.2 ± 0.63	0.32 ± 0.64 (3.5% [−3.4, 10.4])	0.3	1.42 ± 0.64 (15.4% [3.8, 27.0])	5*	1.57 ± 0.94 (36.0% [8.2, 65.8])	2.8
	drought x future conditions	82	0.66	0.66	0	0	6.21	10.76 ± 0.51	-3.65 ± 0.79 (−33.9% [−46.8, −21.1])	21.2***	5.16 ± 0.78 (48.0% [34.8, 61.3])	43.9***	-5.13 ± 1.15 (−33.6% [−62.7, −4.5])	19.8***
	post-drought x future conditions	182	0.18	0.64	0.42	3.61	3.59	8.49 ± 0.69	-0.92 ± 0.74 (−10.8% [−19.9, −1.7])	1.5	2.48 ± 0.70 (29.2% [16.1, 42.2])	12.7***	0.29 ± 1.06 (21.8% [−12.1, 55.7])	0.1
CUE_{sat}	warming x elevated CO_2	241	0.04	0.55	0	0.01	0.01	0.51 ± 0.03	-0.03 ± 0.03 (−5.9% [−13.6, 1.7])	1.1	-0.04 ± 0.03 (−7.8% [−19.4, 3.7])	2.4	-0.01 ± 0.04 (−15.7% [−38.1, 6.7])	0.1
	drought x future conditions	75	0.22	0.49	0	0	0.02	0.41 ± 0.05	0.07 ± 0.06 (17.1% [−11.6, 45.8])	1.4	-0.11 ± 0.06 (−26.8% [−55.5, 1.9])	3.9*	-0.11 ± 0.08 (−36.6% [−92.3, 19.1])	1.6
	post-drought x future conditions	170	0.2	0.56	0	0.01	0.01	0.56 ± 0.03	0.00 ± 0.03 (0.0% [−10.5, 10.5])	0	-0.07 ± 0.03 (−12.5% [−23.0, −2.0])	5.9*	-0.10 ± 0.05 (−30.4% [−53.4, −7.4])	4.5*

Table S3. Sensitivity of Random Forest model performance to complexity (nodesize). Comparison of R^2 values across models for each ecosystem functional property, where nodesize was increased from 5 (default settings, used in the main-text model) to 10 and 20 to reduce model complexity.

Property	*N*	R^2 (nodesize = 5)	R^2 (nodesize = 10)	R^2 (nodesize = 20)	Max. ΔR^2
ET_{sat}	331	0.63	0.63	0.61	–0.02
GPP_{sat}	313	0.42	0.42	0.41	–0.01
WUE_{sat}	288	0.67	0.66	0.65	–0.02
NEP_{sat}	336	0.38	0.38	0.36	–0.02
R_{eco}	336	0.45	0.45	0.45	+0.00
CUE_{sat}	313	0.39	0.39	0.4	+0.01
